# A portable x-ray fluorescence (pXRF) elemental dataset collected from Cambrian-age sandstone aquifer material, Wisconsin, U.S.A.

**DOI:** 10.1016/j.dib.2022.108411

**Published:** 2022-06-23

**Authors:** James J. Zambito, Lisa D. Haas, Michael J. Parsen

**Affiliations:** a Department of Geology, Beloit College, 700 College Street, Beloit, WI 53511 USA; b Department of Geoscience, Lewis G. Weeks Hall for Geological Sciences, University of Wisconsin – Madison, 1215 Dayton Street, Madison, WI 53706, USA; c Wisconsin Geological and Natural History Survey, University of Wisconsin-Madison, 3817 Mineral Point Road, Madison, WI 53705 USA

**Keywords:** Arsenic, Aquifer, Groundwater, Portable x-ray fluorescence, pXRF, Trace metals

## Abstract

This article provides a portable x-ray fluorescence (pXRF) elemental dataset from samples collected from a Cambrian Sandstone Aquifer in West-Central Wisconsin, U.S.A. Analyses were performed on drill core samples and well cutting materials collected using a variety of drilling methods. Elements presented in this dataset include aluminum (Al), arsenic (As), calcium (Ca), cobalt (Co), chromium (Cr), copper (Cu), iron (Fe), potassium (K), magnesium (Mg), manganese (Mn), molybdenum (Mo), nickel (Ni), phosphorus (P), lead (Pb), sulfur (S), silicon (Si), strontium (Sr), uranium (U), vanadium (V), and zinc (Zn). The accuracy and precision of the pXRF analyses was calculated based on repeated measurement of standards of similar lithology to the aquifer. This dataset could be used for 1) chemostratigraphy, 2) refinement of subsurface geochemical sampling techniques; 3) preventing or mitigating naturally-occurring groundwater trace metal contaminants in groundwater in the Upper Mississippi River Valley, and 4) evaluating impacts of regional industrial sand mining on aquifer geochemistry. The data presented in this article was used to select a subset of samples that represented the elemental variability within the overall aquifer succession for further geochemical and mineralogical analysis presented in the article entitled “Identifying the Source of Groundwater Contaminants in West-Central Wisconsin, U.S.A.: Geochemical and Mineralogical Characterization of the Cambrian Sandstone Aquifer” (Zambito et al., 2022).

## Specifications Table

**Table:** 

Subject	Geochemistry and Petrology
Specific subject area	portable x-ray fluorescence (pXRF) elemental analysis of subsurface geological materials
Type of data	TableGraphFigure
How the data were acquired	Elemental analysis was undertaken using a Thermo Fisher Scientific Niton XL3t GOLDD+ Handheld portable X-ray fluorescence (pXRF) analyzer with an 8 millimeter diameter sample window in TestAllGeo Mode; the analyzer was mounted in a lead–lined benchtop stand.
Data format	RawAnalyzedPlotted
Description of data collection	Analyses were performed at the Wisconsin Geological and Natural History Survey (WGNHS, University of Wisconsin - Madison). Well cutting sample material was analyzed in plastic sample cups with a polypropylene film base or in inverted plastic vials using polypropylene film covers. Core sample materials were placed directly on the benchtop stand. pXRF analysis procedures and set-up used herein was based on previously developed WGNHS procedures [2].
Data source location	We analyzed 20 well cuttings sets and 2 drill cores collected in the vicinity of industrial sand mines and the cities of Independence, Whitehall, and Blair in central Trempealeau County, Wisconsin. All of the sample materials analyzed are stored at the Mount Horeb Research Collections and Education Center, operated by the Wisconsin Geological and Natural History Survey (WGNHS, University of Wisconsin - Madison).
Data accessibility	Repository name: EarthChemData identification number: DOI: 10.26022/IEDA/112311Direct URL to data: 10.26022/IEDA/112311Reference: Zambito et al. [3]
Related research article	J.J. Zambito IV, L.D. Hass, M.J. Parsen, Identifying the Source of Groundwater Contaminants in West-Central Wisconsin, U.S.A.: Geochemical and Mineralogical Characterization of the Cambrian Sandstone Aquifer. J. Contam. Hydrol. 247 (2022) 103966. 10.1016/j.jconhyd.2022.103966

## Value of the Data

• These data provide a (spatially) high-resolution elemental characterization of aquifer sandstone in West-Central Wisconsin, U.S.A.
• This dataset will be useful to municipal groundwater resource managers and regulators for understanding baseline aquifer sandstone composition, predicting or mitigating naturally-sourced groundwater quality issues, and identifying potential future groundwater contamination events.
• This dataset could be used for chemostratigraphy, refinement of subsurface geochemical sampling techniques; preventing or mitigating naturally-occurring groundwater trace metal contaminants in groundwater in the Upper Mississippi River Valley, and evaluating impacts of regional industrial sand mining on aquifer geochemistry.

## 1 Data Description

**Fig. 1:**
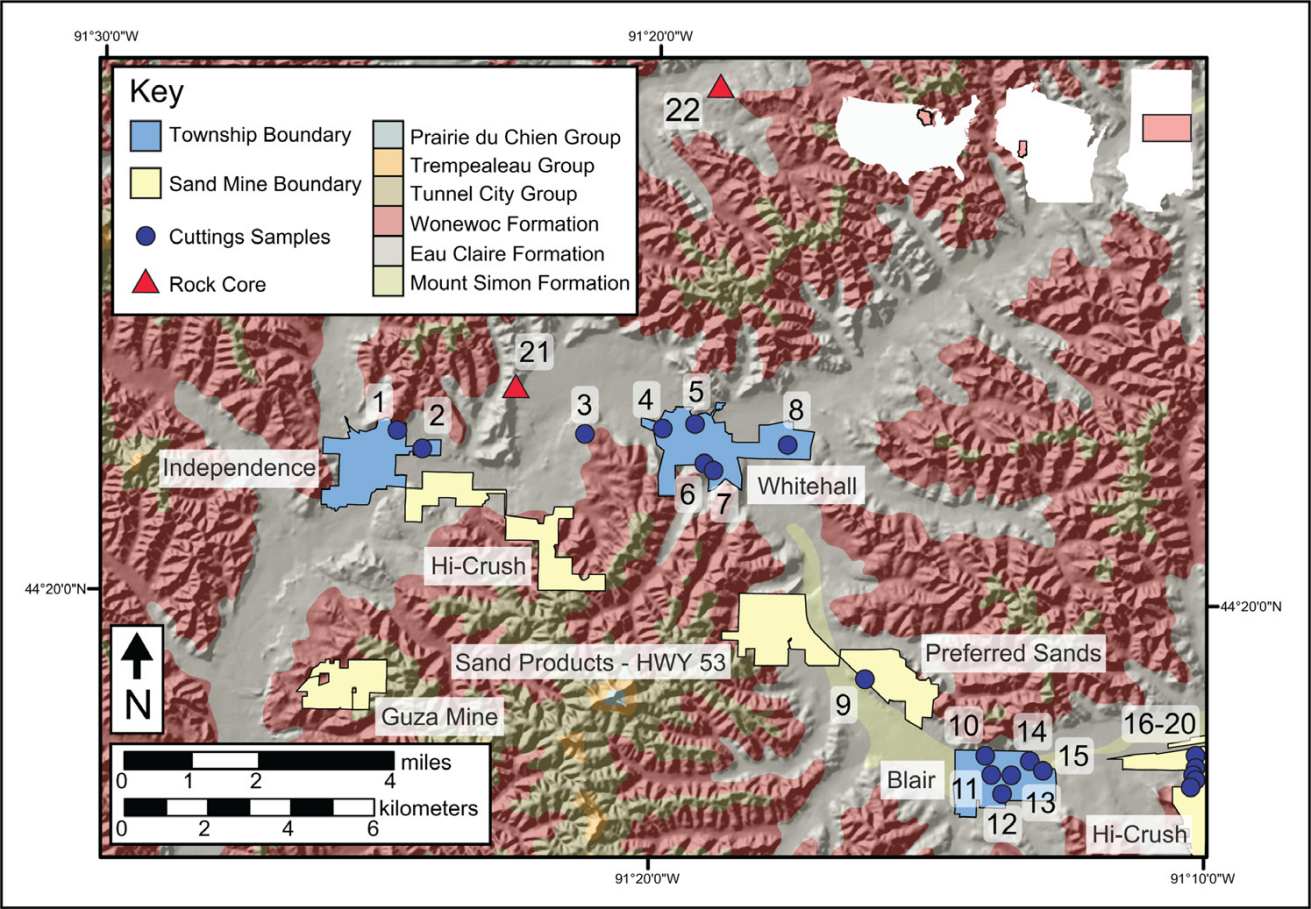
Location of the 22 boreholes studied. Well numbers are the same as used in Table 1. Fig. adapted from [1].

The location of the 22 boreholes studied are shown on the map provided in Fig. 1, which includes the approximate boundaries of municipalities and industrial sand mines for reference. The Fig. 1 basemap is a combination of a hill shade digital elevation model (30-meter resolution) and a bedrock geologic map (created at 1:250,000 scale, [4]). Location details of the study area within the continental U.S.A., State of Wisconsin, and Trempealeau County are shown, from left to right, within the inset white outlines in the upper right region of Fig. 1. Detailed information for each borehole is given in Table 1, keyed to the well number labels used in Fig. 1. In Table 1, Well # refers to the numbers used in Fig. 1, the eight-digit numbers next to well names are Wisconsin Geological and Natural History Survey unique well identifiers (Well ID), curation refers to the storage container for samples from each well, and inferred drilling method is based on age of the well and well construction information available.

**Table 1:** Information on the boreholes and samples studied. Well numbers are the same as used in Fig. 1. Drilling method is given in well construction documentation (see Appendix C) or, when not available, inferred from the age of the borehole.

Well #	Well Name	Year Drilled	Curation	Drilling Method
1	Independence City Test Hole for Well #2 cuttings (62000114)	1997	paper envelope	mud rotary
2	Independence City Well #3 cuttings (62000137)	2009	paper envelope	air rotary
3	Trempealeau County Hospital Well #5 cuttings (62000028)	1957	glass vial	cable tool
4	Land O'Lakes Creamery Well cuttings (62000024)	1953	glass vial	cable tool
5	Whitehall Test Hole #2 cuttings (62000018)	1953	glass vial	cable tool
6	Whitehall City Well #2 cuttings (62000023)	1953	glass vial	cable tool
7	Whitehall City Well cuttings (62000022)	1926	glass vial	cable tool
8	Whitehall City Sewage Treatment Plant Well cuttings (62000065)	1974	old plastic vial	cable tool
9	Winn Bay (Preferred) Sand Well cuttings (62000147)	2011	paper envelope	mud, air, and foam rotary
10	Blair City Well #3 cuttings (62000007)	1945	glass vial	cable tool
11	Associated Milk Producer, Inc. Well #3 cuttings (62000026)	1958	old plastic vial	caisson
12	Blair City Well #4 cuttings (62000045)	1965	old plastic vial	cable tool
13	Blair City Well #5 cuttings (62000068)	1977	old plastic vial	caisson
14	Blair City Test Hole #1-76 cuttings (62000070)	1976	old plastic vial	caisson
15	Blair City Well #6 cuttings (62000109)	1988	paper envelope	caisson
16	Hi-Crush Blair PW-B1 cuttings (62000207)	2015	plastic bag, new plastic vial	mud rotary
17	Hi-Crush Blair PW-B2 cuttings (62000208)	2015	plastic bag, new plastic vial	mud rotary
18	Hi-Crush Blair PW-B4 cuttings (62000209)	2015	plastic bag, new plastic vial	mud rotary
19	Hi-Crush Blair PW-B5 cuttings (62000210)	2015	plastic bag, new plastic vial	mud rotary
20	Hi-Crush Blair PW-B6 cuttings (62000211)	2015	plastic bag, new plastic vial	mud rotary
21	Kulig Quarry core (62000231)	2017	cardboard box	wireline diamond bit, NQ-3
22	Flambeau Mining Co. 96-1-1 core (62000119)	1996	waxed cardboard box	NQ

The entire dataset (a total of 1,177 analyses) of portable X-ray fluorescence (pXRF) elemental analyses collected from these well cutting and drill core samples is presented in Appendix A of the supplementary materials. Also in Appendix A is the data from analysis of standard materials used to determine instrument accuracy and precision (a total of 332 analyses) and the calculations of accuracy and precision (see text below for more details). Elements presented in this dataset include aluminum (Al), arsenic (As), calcium (Ca), cobalt (Co), chromium (Cr), copper (Cu), iron (Fe), potassium (K), magnesium (Mg), manganese (Mn), molybdenum (Mo), nickel (Ni), phosphorus (P), lead (Pb), sulfur (S), silicon (Si), strontium (Sr), uranium (U), vanadium (V), and zinc (Zn). The processed pXRF data is graphed stratigraphically in Appendix B of the supplementary materials; lithologic logs are provided for reference, and discussed in more detail in the companion research paper [1]. We present subsurface depths in the English system of measurement because these cores were drilled in the U.S.A. where subsurface samples are marked in tenths of feet; a metric scale is provided for reference near each lithologic log. Appendix C of the supplementary materials is a compilation of pre-existing geophysical and well construction information for the wells studied.

## 2 Experimental Design, Materials and Methods

### 2.1 Experimental Design

The geochemical and mineralogical data presented herein are focused on the aquitard-aquifer system Cambrian sandstone in the study area, specifically in the vicinity of Whitehall, Wisconsin, the county seat of Trempealeau County (Fig. 1). Some geochemical data for underlying weathered/unweathered crystalline rock and any overlying soil or alluvial/colluvial deposits are also presented. Subsurface samples examined were collected either as drill cuttings or rock core (Table 1), and this diverse set of sample types represents various well construction methods. Since sample type and preparation can influence these analyses and different samples represent different subsurface stratigraphic thicknesses [1,2], a description of the sample materials used, and how they were collected, is included in Table 1. We present subsurface depths in both English and Metric systems of measurement because these cores were drilled in the U.S.A. where subsurface samples are marked in tenths of feet.

### 2.2 Materials

Drill cuttings are the rock pieces and/or ground-up rock powder that are brought to the surface during the boring of a well. Cuttings samples used were collected by three different methods (Table 1). In eight of the older wells, cable tool drilling was used. In this method a bit is raised and dropped, repeatedly chiseling through the bedrock. Periodically, the drill string is removed from the borehole and a bucket-like tool (bailer) is used to collect the drill cuttings, thereby cleaning the borehole and providing a homogenized sample over the depth chiseled. The caisson method, which is essentially a large auger, was used for four of the wells studied that were constructed only into the alluvium/colluvium. The rotary drilling method was used to construct eight wells; in this method, cuttings are brought to the surface by either mud, air, or foam (known as mud, air, and foam rotary drilling, respectively). Mud, air, or foam are used during the drilling process to clean the borehole and cool the spinning drill bit as it grinds through the bedrock. Regardless of method, rotary drill cuttings are typically collected at 5-foot (1.524 m) intervals (though some intervals range from 2 to 10 feet, or 0.6096 to 3.048 m, in the boreholes studied). In rotary and caisson drilling, each cuttings sample is some representation of a 5-foot (1.524 m) interval of drilled rock; typically, a ‘grab sample’ was collected while that 5-foot (1.524 m) interval was drilled. This is in contrast to wells drilled by cable tool, which are more likely a homogenized sample over the depth interval sampled.

In contrast to cuttings, drill core samples are a continuous representation of the subsurface, and provide a more complete geologic representation of this aquitard-aquifer system [5]. Rock core drilling uses a hollow drill bit that results in a continuous cylindrical rock sample, and uses drilling mud to keep the drill bit cool while grinding away the bedrock. After the core is removed from the core barrel, it is ideally washed with potable water to clean off any drilling mud. Unlike cuttings, rock core can be studied and sampled at discrete depths of a tenth to a hundredth of a foot (∼3 to 0.3 cm) in order to capture lithologic heterogeneity. Furthermore, drill core allows us to better understand sedimentary structures and mineral morphologies that are key to understanding the association of trace metal concentrations with mineral phases, and the origin of any trace metal-bearing, redox sensitive mineralization present.

We analyzed 20 well cuttings sets and 2 drill cores in the vicinity of industrial sand mines and the cities of Independence, Whitehall, and Blair in central Trempealeau County, Wisconsin (Fig. 1, Table 1). The majority of the samples were collected prior to large-scale industrial sand mining in the region, with the oldest dating back to 1926 (Table 1). For some well cuttings sets, samples were not collected from all depths of the borehole, and some intervals of drill core are missing due to a lack of recovery of friable sandstone during the coring process. All of the sample materials analyzed are housed at the Mount Horeb Research Collections and Education Center, operated by the Wisconsin Geological and Natural History Survey (WGNHS, University of Wisconsin - Madison).

### 2.3 Methods

Elemental analysis by pXRF was undertaken at the WGNHS using a Thermo Fisher Scientific Niton XL3t GOLDD+ Handheld portable X-ray fluorescence (pXRF) analyzer with an 8 millimeter diameter sample window; the analyzer is mounted in a lead–lined benchtop stand. Each pXRF analysis took a total of 90 s (Filter settings: 30 s Main filter, 30 s Light filter, and 30 s Low filter; different filters measure different suites of elements, the High filter was not used) and was performed in TestAllGeo Mode. The reader is referred to Zambito and others [2] for further details on WGNHS pXRF analysis instrumentation procedures and set-up.

The well cuttings analyzed were collected and curated over a span of 89 years (Table 1) and, correspondingly, drilling and curation methods as well as storage containers varied. Sample preparation methods prior to pXRF analysis varied slightly with the goal of performing minimal sample preparation (see Zambito and others [2], for pictures and description of these preparation techniques outlined below). Well cuttings that were curated prior to 1957 are stored in glass vials; since the silicon in the glass could be detected by the pXRF, we analyzed these samples in a plastic sample cup with a polypropylene film base that is 4.0 microns (0.16 mil) thick. Well cuttings that were curated between 1958 and 1977 are stored in older clear plastic vials. Since the major components of the plastic (carbon, hydrogen, and oxygen) cannot be detected by the pXRF, the cuttings were kept in their plastic vials and we used dental rubber bands (the type used on braces) to secure polypropylene film (4.0 micron thick) around the mouth of the plastic vial. We then analyzed the cuttings samples by inverting the plastic vial on the pXRF sample stage so that the sample was analyzed through the polypropylene film covering the mouth of the vial. Well cuttings curated since 1988 are stored in paper envelopes; similar to samples stored in glass vials, we analyzed these samples in plastic sample cups with a polypropylene film base (4.0 microns thick). The most recently obtained cuttings, collected during the construction of groundwater and monitoring wells at the Hi-Crush industrial sand mine near Blair, were obtained at the time this project was being undertaken and had not yet been curated into standard paper envelopes. We therefore subsampled the well cuttings (provided by the drillers in plastic bags), placing the cuttings material in new plastic vials. Similar to cuttings in clear plastic vials curated between 1958 and 1977, we secured polypropylene film (4.0 microns thick) to the vial mouths, and inverted the vials on the pXRF sample stage for analysis.

The polypropylene film used in our analyses was also analyzed using the pXRF instrument without any sample; the analysis run immediately failed indicating that no elemental concentrations from the polypropylene film were detected by the instrument itself using the Main filter. The Main filter, which analyzes for Ag, As, Au, Bi, Cd, Co, Cr, Cu, Fe, Hf, Hg, Mn, Mo, Nb, Ni, Pb, Pd, Rb, Re, Sb, Se, Sn, Sr, Ta, Th, Ti, U, V, W, Zn, and Zr, is the first to be run. However, film impurities do occur at trace levels [6].

For rock core analysis, core samples were sanded with 60 to 100 grit sanding disks where pXRF analysis would be undertaken to remove any drilling mud residue or secondary minerals (for example, oxides) that may have formed on the core surface since it was drilled. Core samples were then meticulously cleaned with a compressed-air duster and placed directly on the sample stage with the sanded spot over the pXRF analyzer 8 millimeter diameter sample window. The pXRF sample window size allows for discrete sampling of the lithologic heterogeneity exhibited in the rock cores [2]. Although no polypropylene film was used for rock core samples, the pXRF instrument itself uses a Prolene® (registered trademark of Chemplex) film to protect its sensors from contamination, for example sand grains that might fall off of a piece of rock core and into the instrument. Similar to the polypropylene film mentioned above, this Prolene film is also undetectable to the pXRF instrumentation when analyzed by itself (see Wilson and others [6], for further details of XRF Prolene film composition and physical properties).

### 2.4 Data Processing: Accuracy and Precision

The precision and accuracy of the pXRF instrument was determined for each element of interest using the instrument results for repeated analysis of standard materials (180-647, SBC-1, SDO-1, SRM-1d; details are found in Appendix A; see also Rowe and others [7], Knight and others [8], and Stewart and Mauk [9]). Each standard was measured at least 80 times over the course of data collection for the 20 cuttings sets and 2 cores, typically at the beginning, middle, and end of a pXRF data collection session (Appendix A). In order to determine accuracy and precision of the instrument, and any differences in accuracy and precision that may exist among different lithologies (matrices), it is necessary to use standards similar to the materials being analyzed when calculating calibration factors [7,8]. We determined instrument contamination, accuracy, and precision as well as XRF film impurities by repeated measurement of the standard materials representing a SiO2 ‘blank’, two types of shales, and an argillaceous limestone (see below and Appendix A). Precision as discussed herein is determined using relative standard deviation; we consider the measurement of an element to be precise if the relative standard deviation is <= 20%. The smaller the relative standard deviation, the more precise the data [8]. Accuracy is determined by how close the mean measurement of measured element concentration is to the accepted value by calculating the percent error of the mean. We consider the measurement of an element to be accurate and quantitatively meaningful if the percent error of the mean is <= +/-20% [9]. Finally, we note elements that are problematic when measured by pXRF (see below).

A SiO2 standard (180-647, provided by Thermo Fisher Scientific with the pXRF instrument used) was used as a ‘blank’ in order to determine the trace metal elemental concentrations present as either impurities in the XRF films used, or instrument contamination. As shown in Appendix A, the instrument output for As, Co, Cr, Cu, Mn, Ni, Pb, U, or Z when analyzing this standard was that concentrations were less than the limit of detection. Concentrations of Mo and V detected fall within the accepted values for this standard. The instrument detected concentrations of Al, Ca, Fe, K, Mg, and Sr that are greater than the accepted concentrations (<10 ppm) for this standard. However, relative to the concentrations present in the majority of rocks studied, these impurities are in trace concentration. Concentrations of S determined by pXRF are higher than the accepted values and at the lower-end of the rocks studied suggesting pXRF measurements of this element may be problematic. Furthermore, the instrument detected high concentrations of P and V relative to the concentrations present in the majority of rocks studied; this suggests that interpretation of pXRF measurement of P and V is problematic (Knight and others [8] also found V pXRF data interpretation problematic). Finally, the instrument underestimated the concentration of silicon in this standard.

The standards USGS SBC-1 and SDO-1 were used to understand the accuracy and precision of the pXRF instrument when analyzing a mudstone. SBC-1 (Brush Creek Shale) is a marine shale from the Pennsylvanian Glenshaw Formation at Greensburg, Pennsylvania with major concentrations of muscovite, quartz, kaolinite, and chlorite with minor concentrations of calcite, siderite, anatase, rutile, and pyrite. This lithology is somewhat similar to that of the Eau Claire Formation of the analyzed samples, but less sandy and non-glauconitic. SDO-1 (Devonian Ohio Shale) is a black, organic- and sulfur-rich shale from the Devonian Ohio Shale at Morehead, Kentucky that is relatively enriched in trace metals. Additionally, we used the NIST standard SRM-1d, which is an argillaceous limestone from Putnam County, Indiana; this standard is somewhat lithologically similar to portions of the Eau Claire Formation, though the carbonate cement in the Eau Claire is dolomitic. The results of pXRF analysis of these standards is presented in Appendix A.

Based on the analysis of standards, the pXRF-determined measurements of Ca, Cu, Pb, and Sr are all precise and accurate. Determination of Si and Zn is also precise and accurate using the shale standards, though for the argillaceous limestone standard (SRM-1d) the pXRF results were imprecise for Si and were inaccurate for Zn. Determination of As returned many results that were less than the limit of detection (< LOD) for the argillaceous limestone standard suggesting it may be problematic, though elemental concentrations for As, Co, Cu, Mo, and Pb were not available for the argillaceous limestone standard. Interpreting Mg measurements is possible at higher concentrations: it is precise and accurate at a concentration of 1.56%, imprecise but accurate at a concentration of 9286.84 ppm, and precise but inaccurate (with many < LOD returned) at a concentration of 1815.16 ppm. This may be attributable to the fact the Mg is the lightest element detectable to the pXRF. Likewise, Mn measurements are accurate but not always precise at concentrations of 325.27 and 1161.65 ppm, but inaccurate at a concentration of 161.86 ppm. Measurements of Mo are precise, but more accurate at higher concentrations (134.0 ppm versus 2.4 ppm). Conversely, Sr measurements are precise, but more accurate at lower concentrations (1028.0 and 7150.0 ppm versus 5.35%). The concentrations of Al, Fe, and K are generally precise, but inaccurate in that they consistently overestimate concentration two-fold (these elements can be calibrated however [2,7]). Similarly, Ni measurements by pXRF are precise but two-fold or more overestimate the true concentration, making interpretation of this element complicated. Measurements of U are precise and accurate at a concentration of 48.8 ppm, but problematic at concentrations of 1.0 and 5.76 ppm because at these low concentrations the instrument measurement is either an overestimation of the true value or returned as < LOD. Lastly, Co, Cr, P and V measurements are never accurate based on the standards used herein, and furthermore, either overestimate the true value or returned as < LOD.

Based on this analysis of pXRF standard precision and accuracy (Appendix A), we determined that data for Cr, Co, Ni, P, and V were likely not accurate and therefore pXRF data for these elements is not plotted (Appendix B). In contrast, based on the determined pXRF precision and accuracy (Appendix A), the concentrations of Al, As, Ca, Cu, Fe, K, Mg, Mn, Mo, Pb, S, Si, Sr, U, and Zn are plotted (Appendix B). Although pXRF measurements were found to be inaccurate for Al, K, and Fe, these are included in the graphical plots because they are major rock-forming elements and qualitatively useful for chemostratigraphic correlation and inferring lithology [5,10].

### 2.5 Graphical Data Presentation

All elemental data collected by pXRF are presented graphically (plotted) in Appendix B. For plotting pXRF elemental data from cuttings, we chose the mid-point of the depth range; for example, data from a cuttings sample from 210 to 215 feet (64.0 to 65.5 m) was plotted at a depth of 212.5 feet (64.77 m). Elemental data from cores were plotted at the depth that the samples were taken. Values that were less than the limit of detection (< LOD) of the pXRF instrument were converted to the value zero for plotting purposes; LOD is determined by the instrument based on the testing mode used. The data plotted are the measurements returned by the pXRF analyzer; these data have not been calibrated and the error bars shown for pXRF measurements are the instrument's uncertainty in the measurement and not the error based on instrument accuracy. In all plots of pXRF elemental data, we scaled well depths and (for the most part) elemental concentrations similarly among boreholes so that data from different wells could be easily compared. Some elements had consistently higher concentrations in core than cuttings samples, and elemental concentration scales between these sample types differ accordingly. Furthermore, for consistency and ease of comparison among geochemical plots, if no accurate and precise data for a given element is available (see data processing above, Appendix B) the scale is still present but partially transparent. We graphically present pXRF elemental data for elements that are known to cause adverse health effects and for which the pXRF instrument is precise and accurate (As, Cu, Pb, Mn, Mo, Sr, U, and Zn) as well as elements that can be used to identify subsurface stratigraphic units (Al, Ca, Fe, K, Mg, Si, and S; see discussion in Zambito and others [2,5,10]).

## Ethics Statements

Not applicable; no animals or humans were analyzed.

## CRediT authorship contribution statement

**James J. Zambito:** Conceptualization, Funding acquisition, Methodology, Investigation, Data curation, Formal analysis, Writing – original draft. **Lisa D. Haas:** Methodology, Investigation, Data curation, Writing – review & editing. **Michael J. Parsen:** Conceptualization, Funding acquisition, Writing – review & editing.

## Declaration of Competing Interest

The authors declare that they have no known competing financial interests or personal relationships which have, or could be perceived to have, influenced the work reported in this article.

## Data Availability

A portable x-ray fluorescence (pXRF) elemental dataset collected from Cambrian-age sandstone aquifer material, Wisconsin, U.S.A. (Original data) (EarthChem).

## Appendix Supplementary materials

Image, application 1
