# Supplementary material for: A portable x-ray fluorescence (pXRF) elemental dataset collected from Cambrian-age sandstone aquifer material, Wisconsin, U.S.A
Source: Data Brief. 2022 Jun 23;43:108411. doi: 10.1016/j.dib.2022.108411 (PMC9251326; doi:10.1016/j.dib.2022.108411)
Supplement: Supplementary file 1 [file mmc1.zip › Appendix B_Graphical representation of pXRF data.pdf]

A portable x-ray fluorescence (pXRF) elemental dataset collected from Cambrian-age sandstone aquifer material, Wisconsin, U.S.A.

James J. Zambito IV, Lisa D. Haas, and Michael J. Parsen

zambitoj@beloit.edu

Appendix B

This appendix contains the plotted pXRF elemental data for each of the wells studied. Elemental concentrations that are higher than the scale used are denoted by asterisks and the determined concentration is listed (see Appendix A of supplemental materials for full pXRF dataset). For elemental data, if error bars are not visible, then they are smaller than the size of the data point used.

Each figure shows a lithologic log for the well, down-hole natural gamma radiation log (if available) and well construction information (if available), the latter including the depth of well casing (black lines) and water table level (blue area); see Appendix C of supplemental materials for a compilation of pre-existing geophysical and well construction data.

Well 1 - Independence Test Hole for Well #2 (62000114)

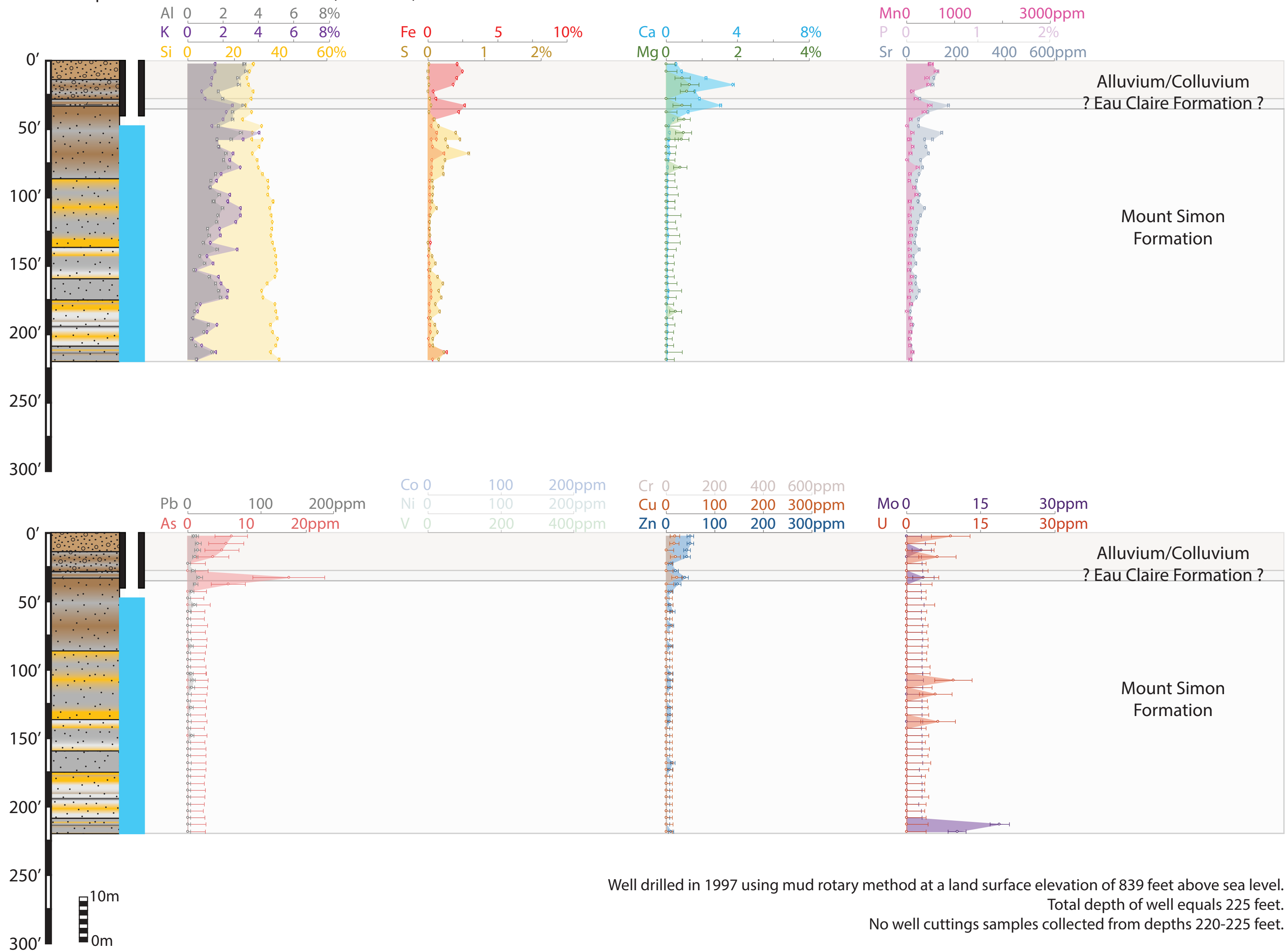

Well drilled in 1997 using mud rotary method at a land surface elevation of 839 feet above sea level.
Total depth of well equals 225 feet.
No well cuttings samples collected from depths 220-225 feet.

Well 2 - Independence City Well #3 (62000137)

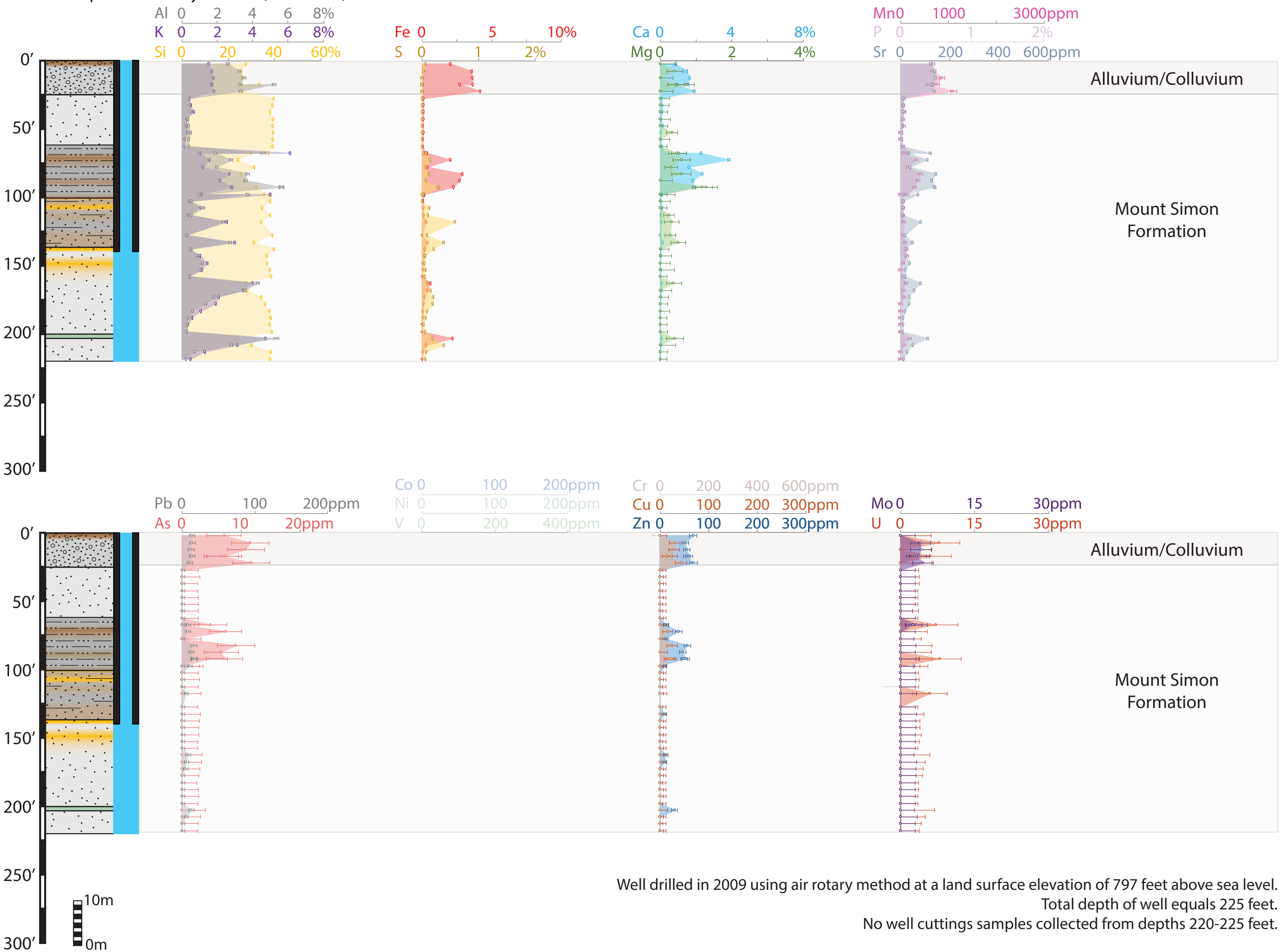

Well drilled in 2009 using air rotary method at a land surface elevation of 797 feet above sea level.
Total depth of well equals 225 feet.
No well cuttings samples collected from depths 220-225 feet.

Well 3 - Trempealeau County Hospital Well #5 (62000028)

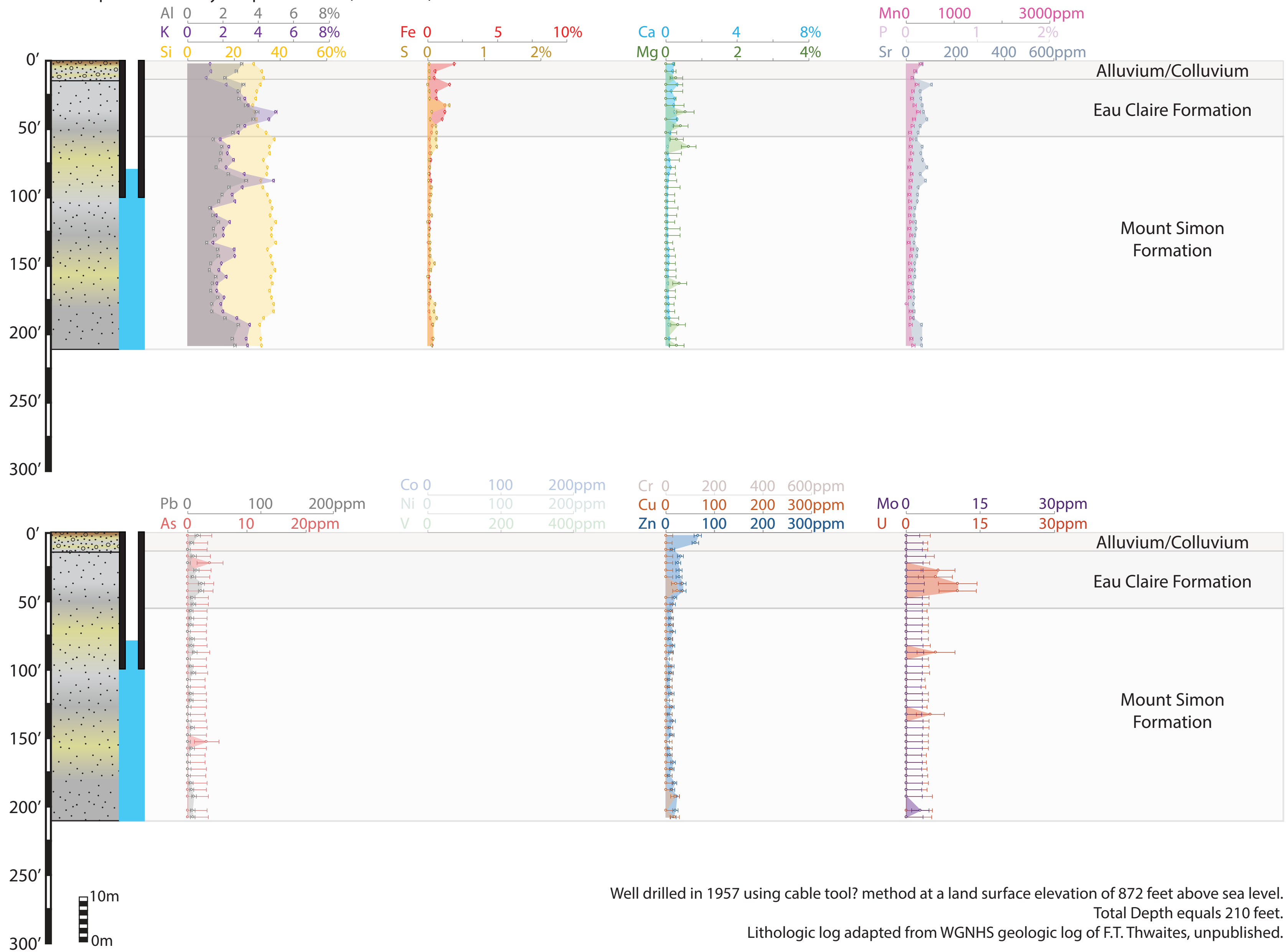

Well drilled in 1957 using cable tool? method at a land surface elevation of 872 feet above sea level.
Total Depth equals 210 feet.
Lithologic log adapted from WGNHS geologic log of F.T. Thwaites, unpublished.

Well 4 - Land O' Lakes Creamery Well #2 (62000024)

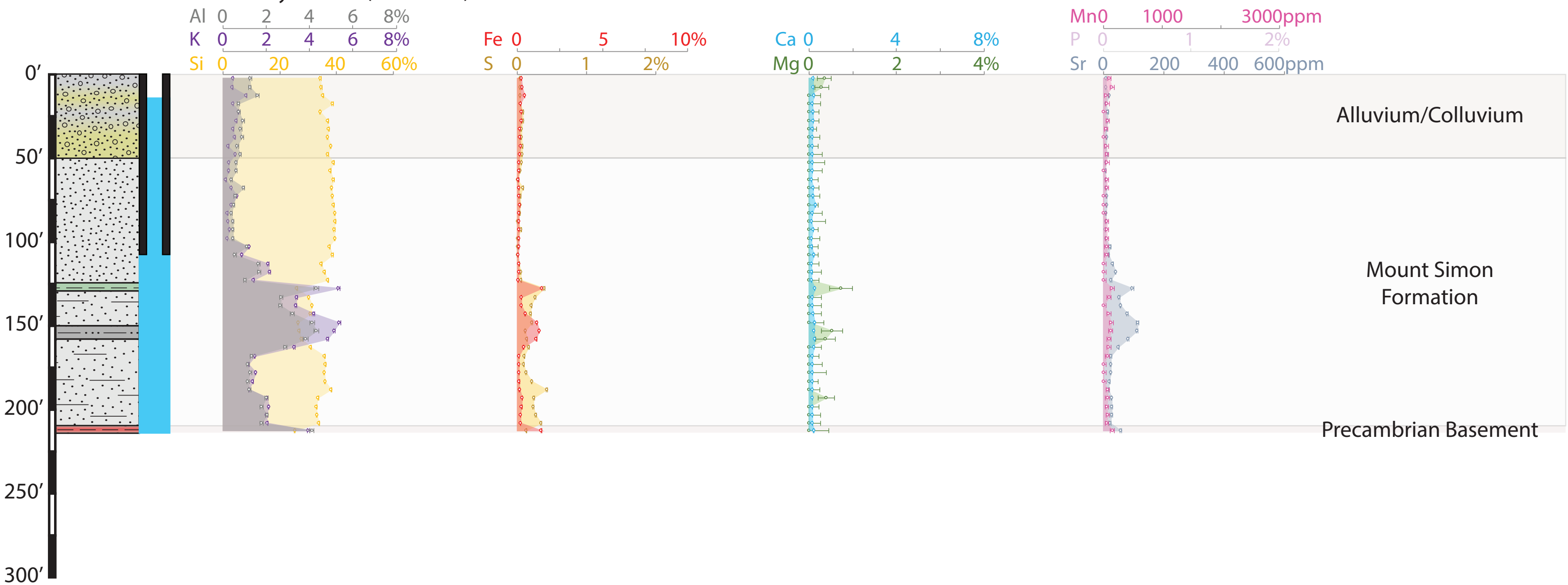

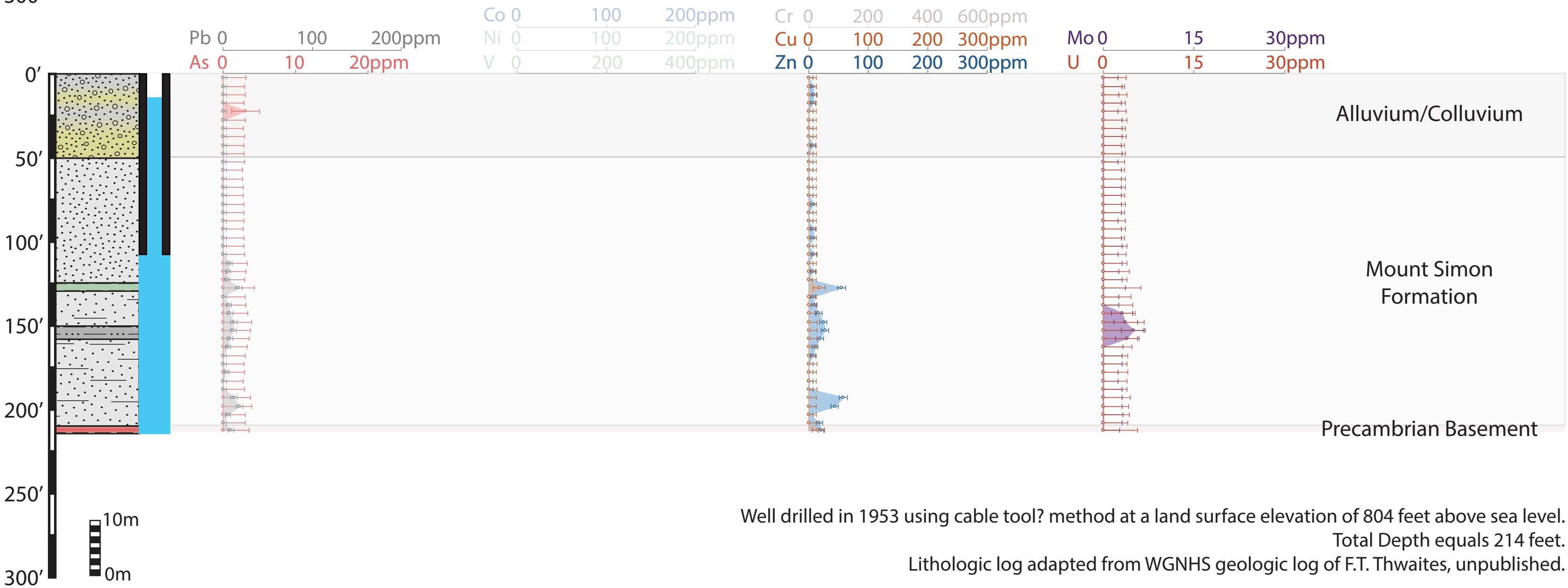

Well drilled in 1953 using cable tool? method at a land surface elevation of 804 feet above sea level.
Total Depth equals 214 feet.
Lithologic log adapted from WGNHS geologic log of F.T. Thwaites, unpublished.

Well 5 - Whitehall Test Hole #2 (62000018)

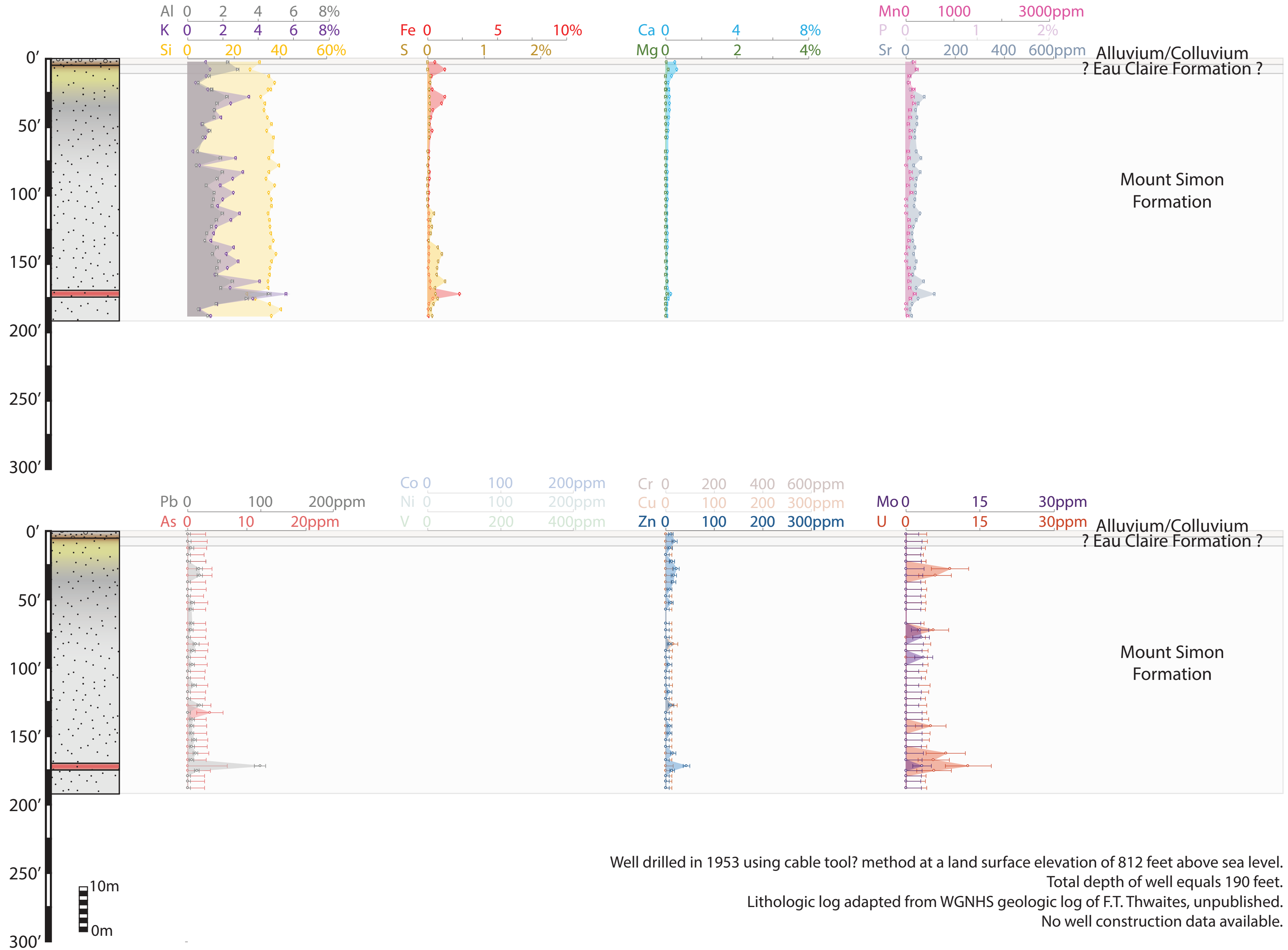

Well drilled in 1953 using cable tool? method at a land surface elevation of 812 feet above sea level.
Total depth of well equals 190 feet.
Lithologic log adapted from WGNHS geologic log of F.T. Thwaites, unpublished.
No well construction data available.

Well 6 - Whitehall City Well #2 (62000023)

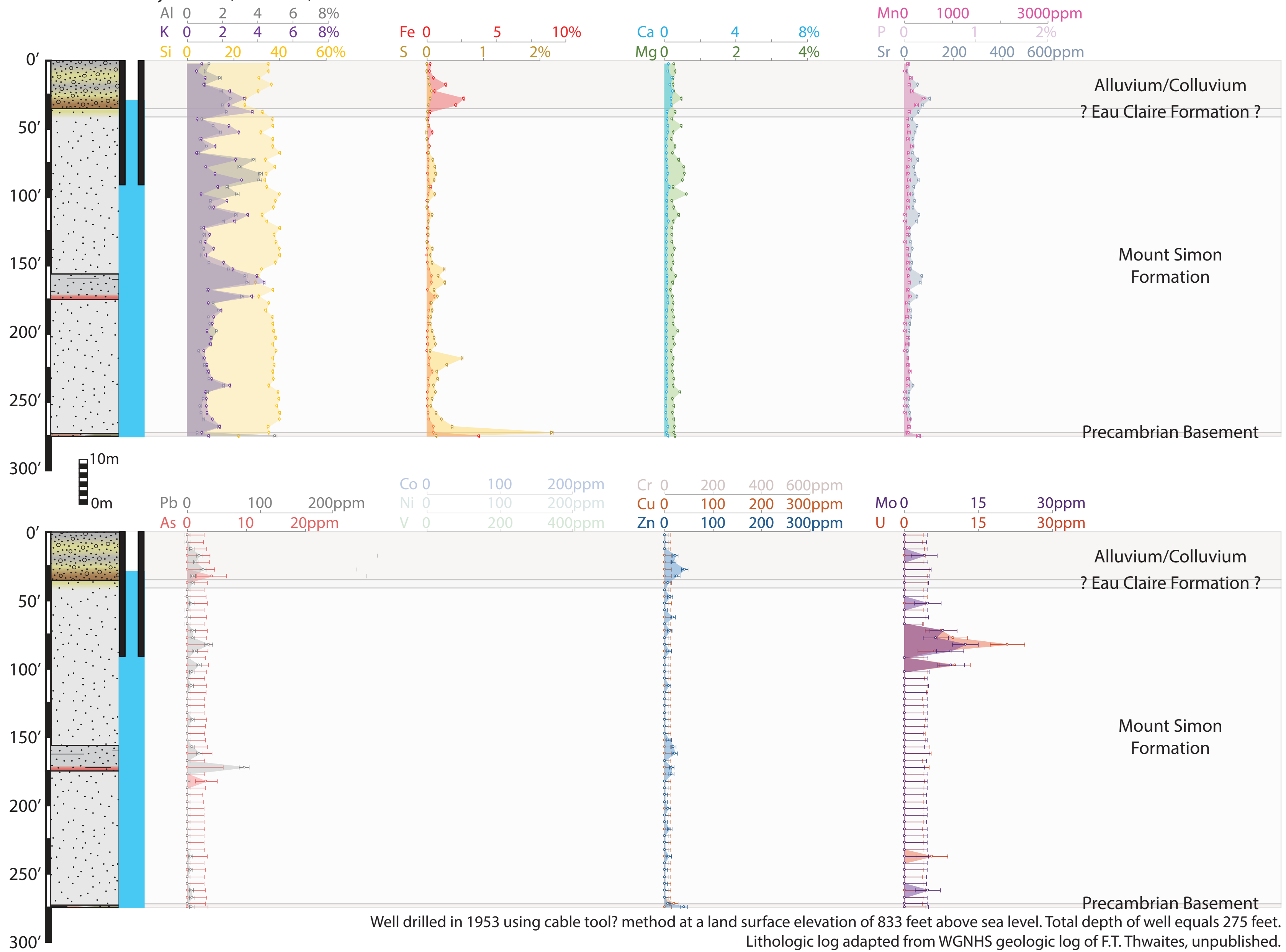

Well drilled in 1953 using cable tool? method at a land surface elevation of 833 feet above sea level. Total depth of well equals 275 feet.
Lithologic log adapted from WGNHS geologic log of F.T. Thwaites, unpublished.

Well 7 (part a) - Whitehall City Well (62000022)

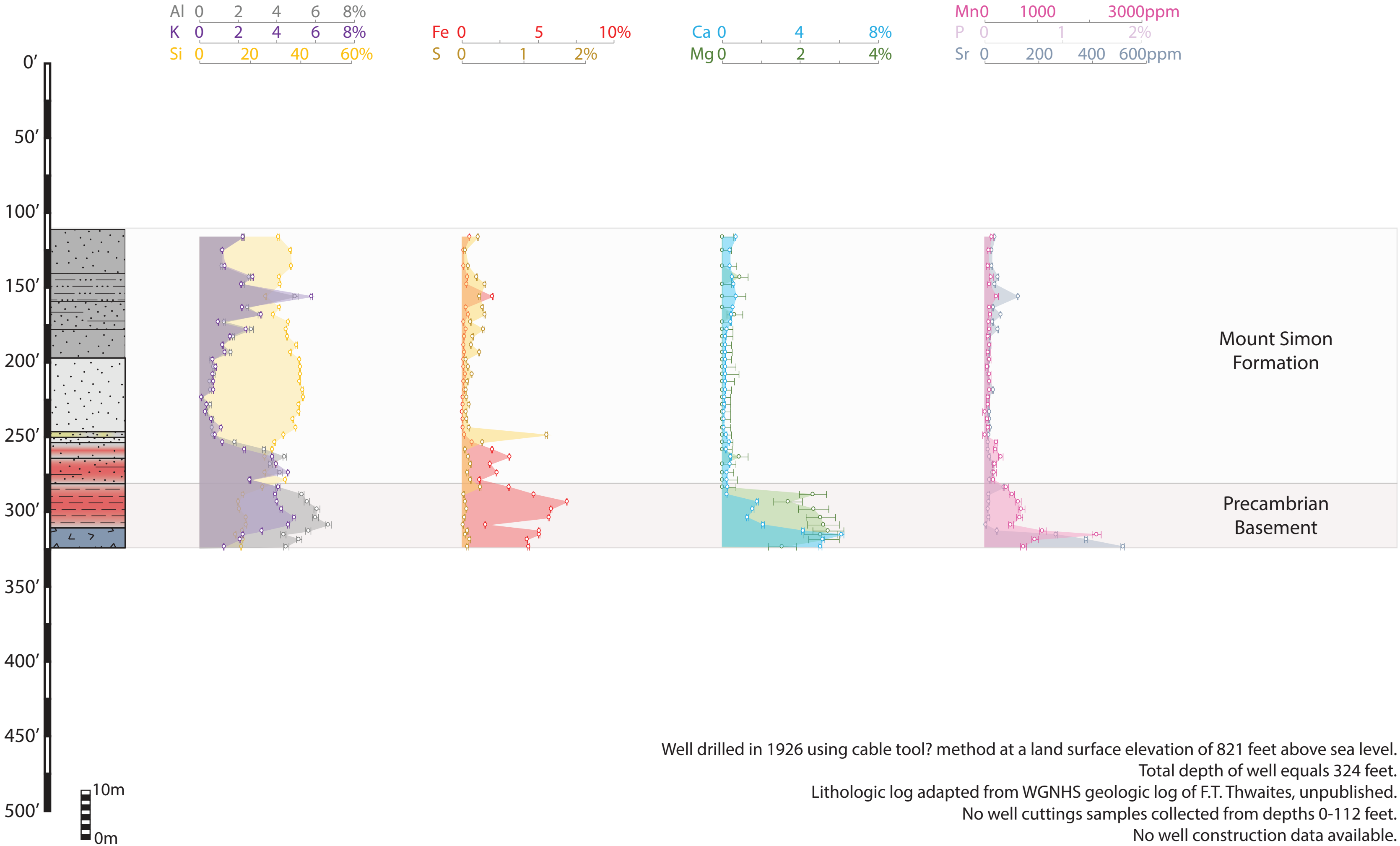

Well drilled in 1926 using cable tool? method at a land surface elevation of 821 feet above sea level.
Total depth of well equals 324 feet.
Lithologic log adapted from WGNHS geologic log of F.T. Thwaites, unpublished.
No well cuttings samples collected from depths 0-112 feet.
No well construction data available.

Well 7 (part b) - Whitehall City Well (62000022)

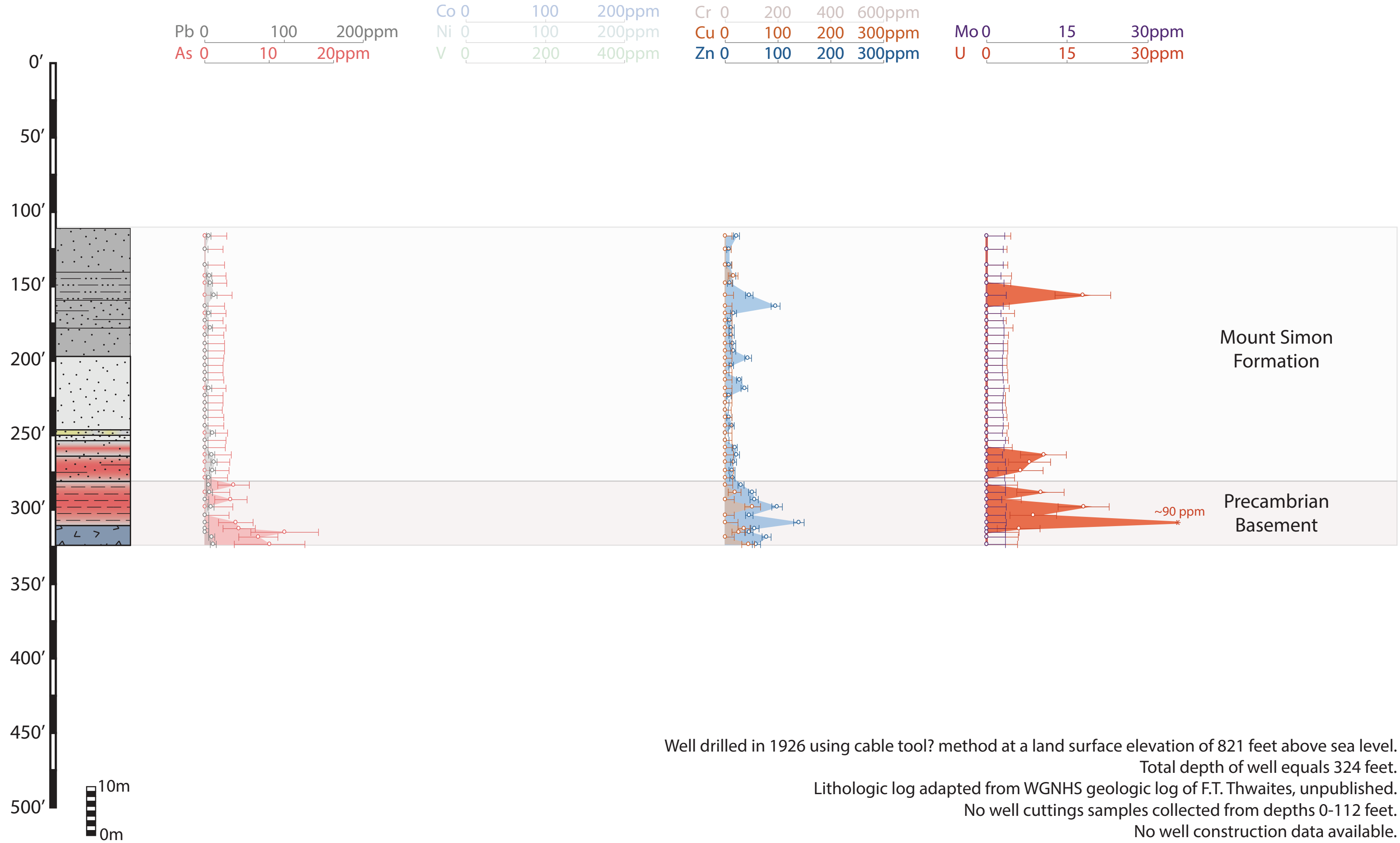

Well drilled in 1926 using cable tool? method at a land surface elevation of 821 feet above sea level.
Total depth of well equals 324 feet.
Lithologic log adapted from WGNHS geologic log of F.T. Thwaites, unpublished.
No well cuttings samples collected from depths 0-112 feet.
No well construction data available.

Well 8 - Whitehall Sewage Treatment Well (62000065)

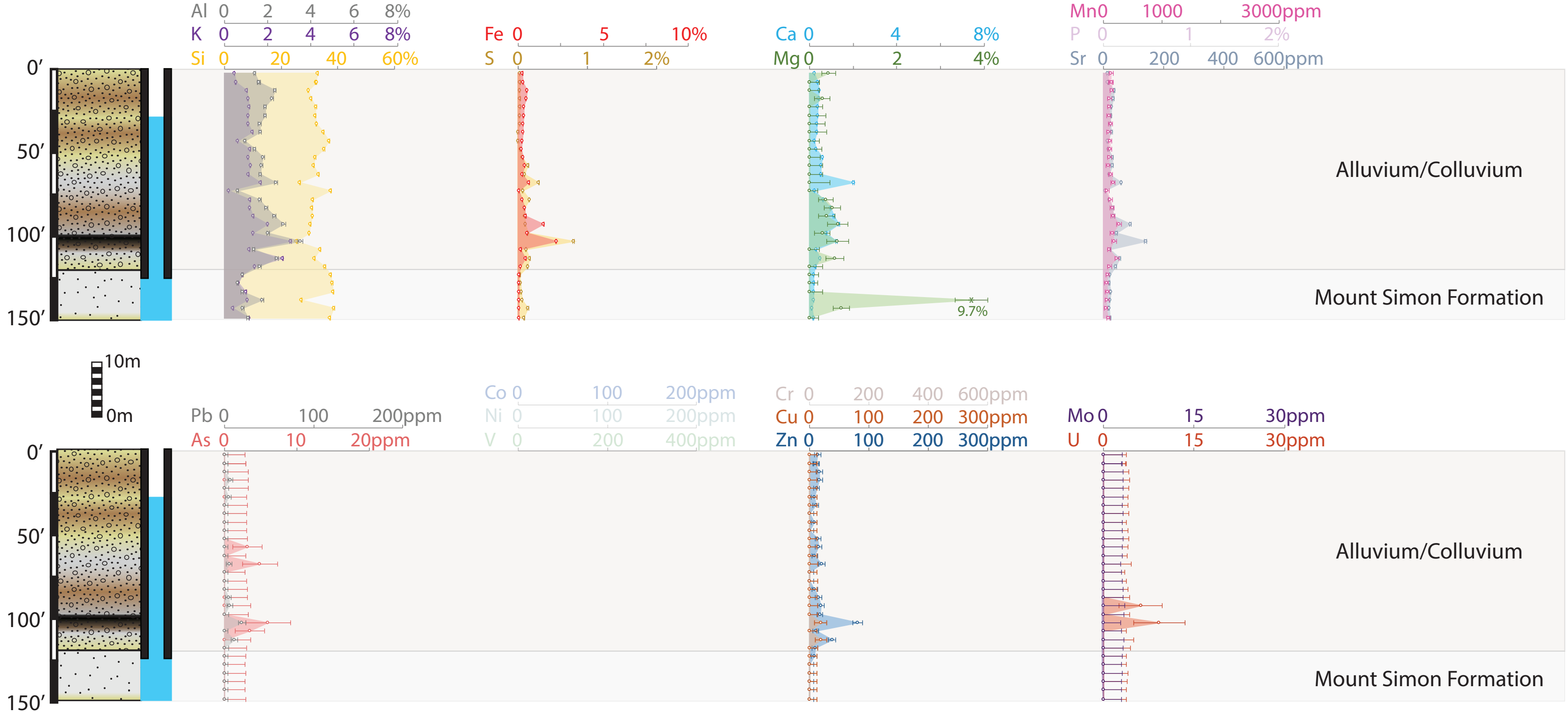

Well drilled in 1974 using cable tool method at a land surface elevation of 838 feet above sea level. Total depth of well equals 150 feet.
Lithologic log adapted from WGNHS geologic log of R.M. Peters, unpublished.

Well 9 (part a) - Winn Bay (Preferred) Sand Well (62000147)

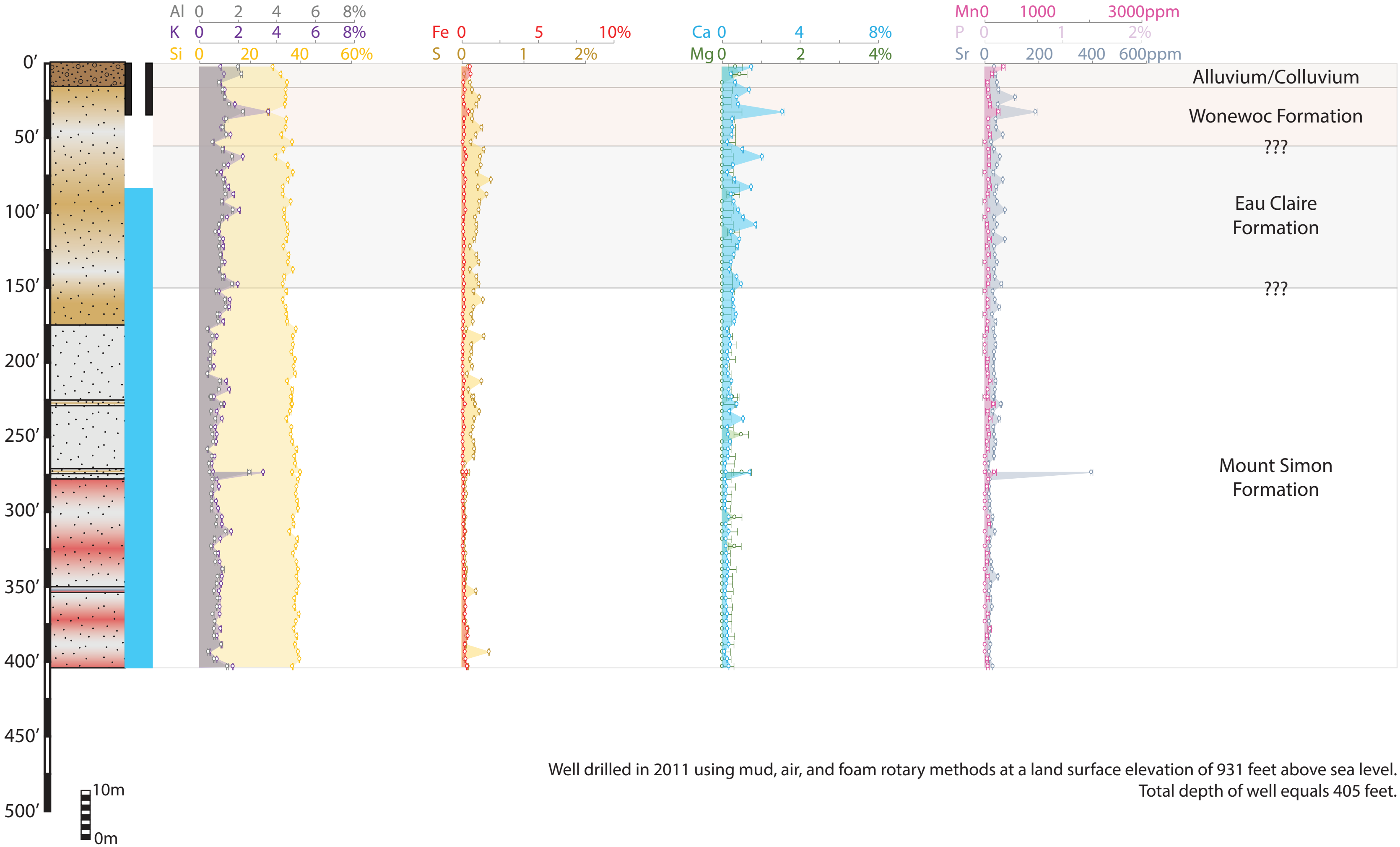

Well drilled in 2011 using mud, air, and foam rotary methods at a land surface elevation of 931 feet above sea level.
Total depth of well equals 405 feet.

Well 9 (part b) - Winn Bay (Preferred) Sand Well (62000147)

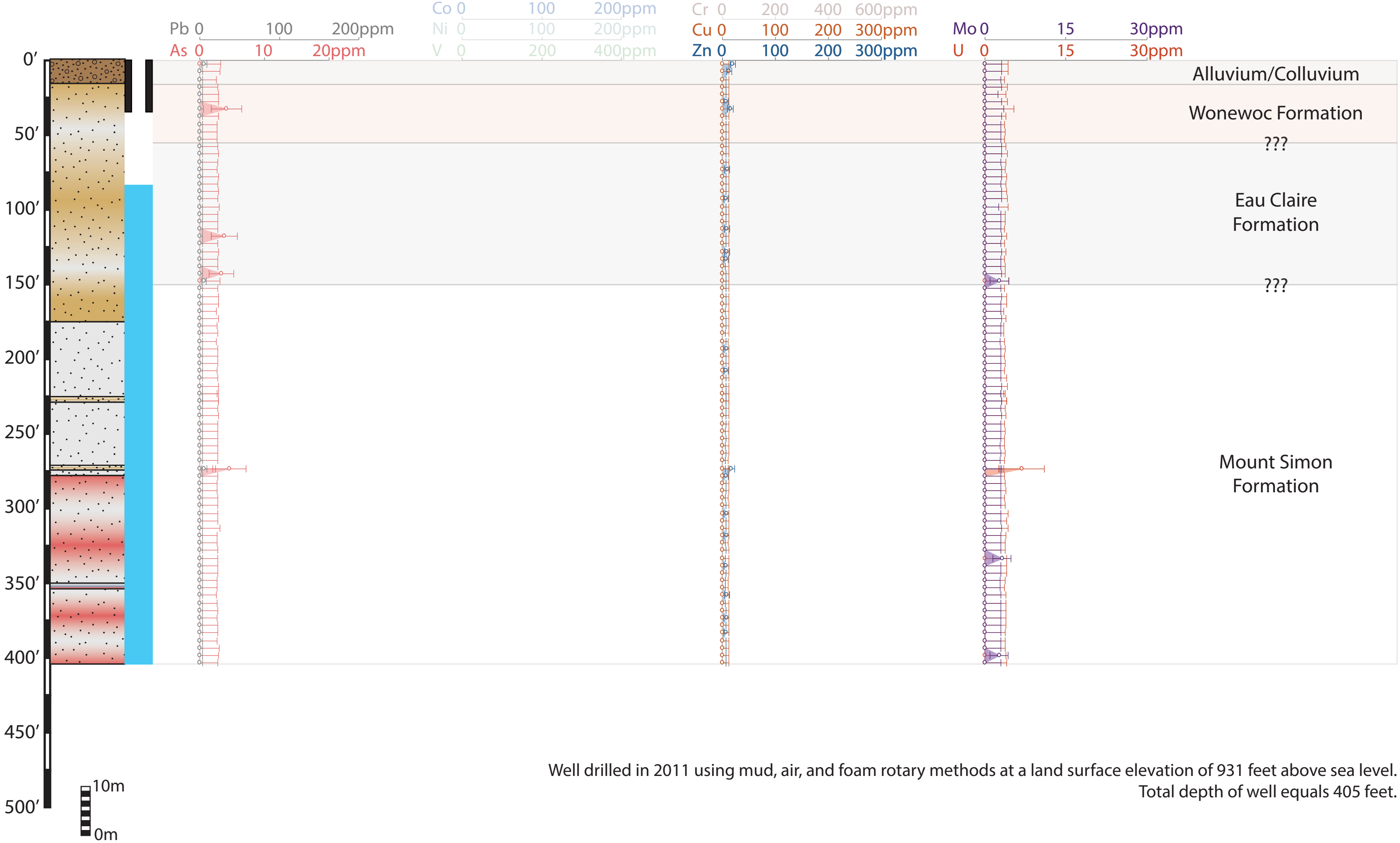

Well drilled in 2011 using mud, air, and foam rotary methods at a land surface elevation of 931 feet above sea level.
Total depth of well equals 405 feet.

Well 10 - Blair City Well #3 (62000007)

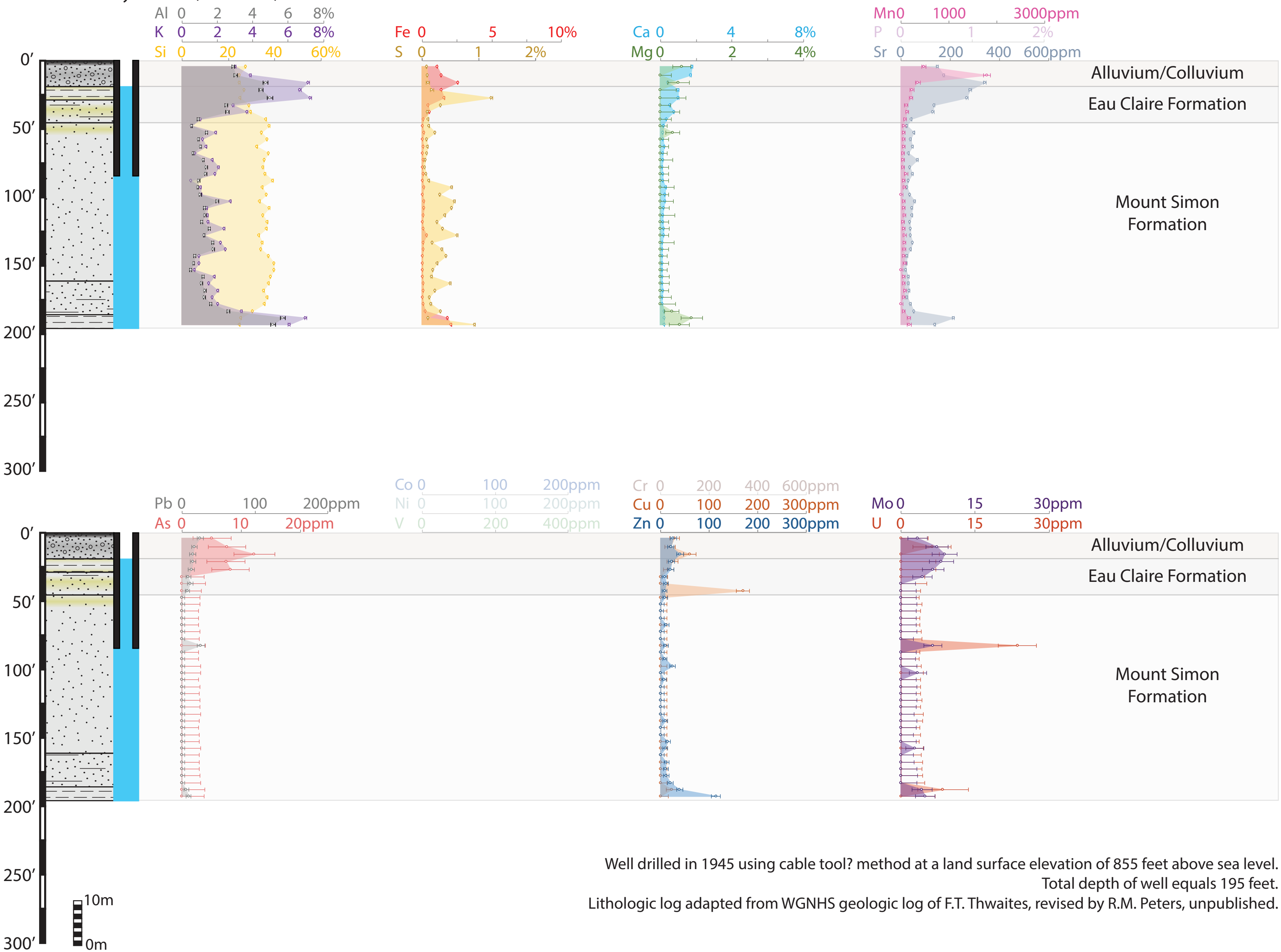

Well drilled in 1945 using cable tool? method at a land surface elevation of 855 feet above sea level.
Total depth of well equals 195 feet.
Lithologic log adapted from WGNHS geologic log of F.T. Thwaites, revised by R.M. Peters, unpublished.

Well 11 - Associated Milk Producers Well #3 (a.k.a. Preston Creamery, Western Wisconsin Dairies Coop) (62000026)

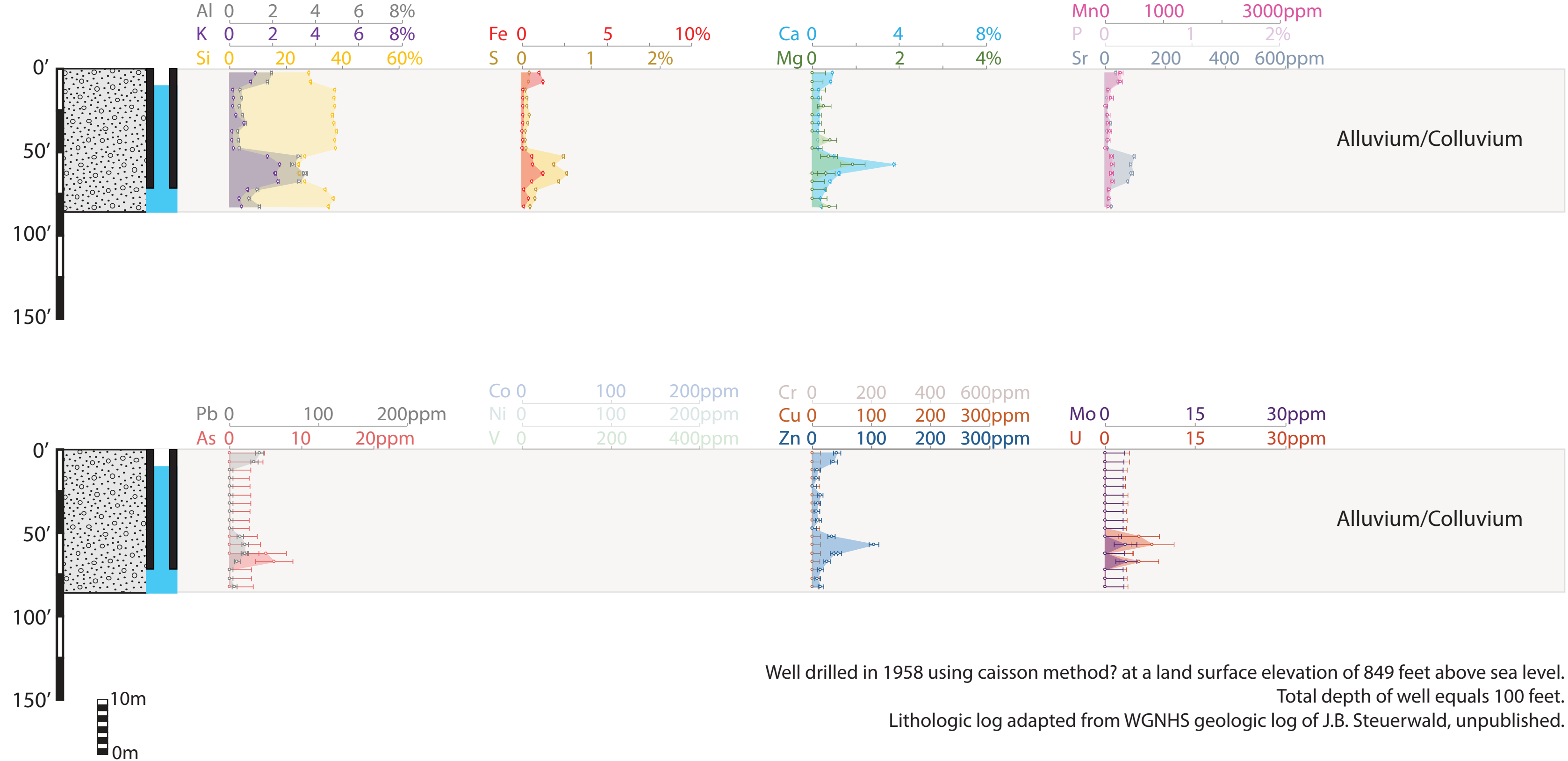

Well drilled in 1958 using caisson method? at a land surface elevation of 849 feet above sea level.
Total depth of well equals 100 feet.
Lithologic log adapted from WGNHS geologic log of J.B. Steuerwald, unpublished.

Well 12 - Blair City Well #4 (62000045)

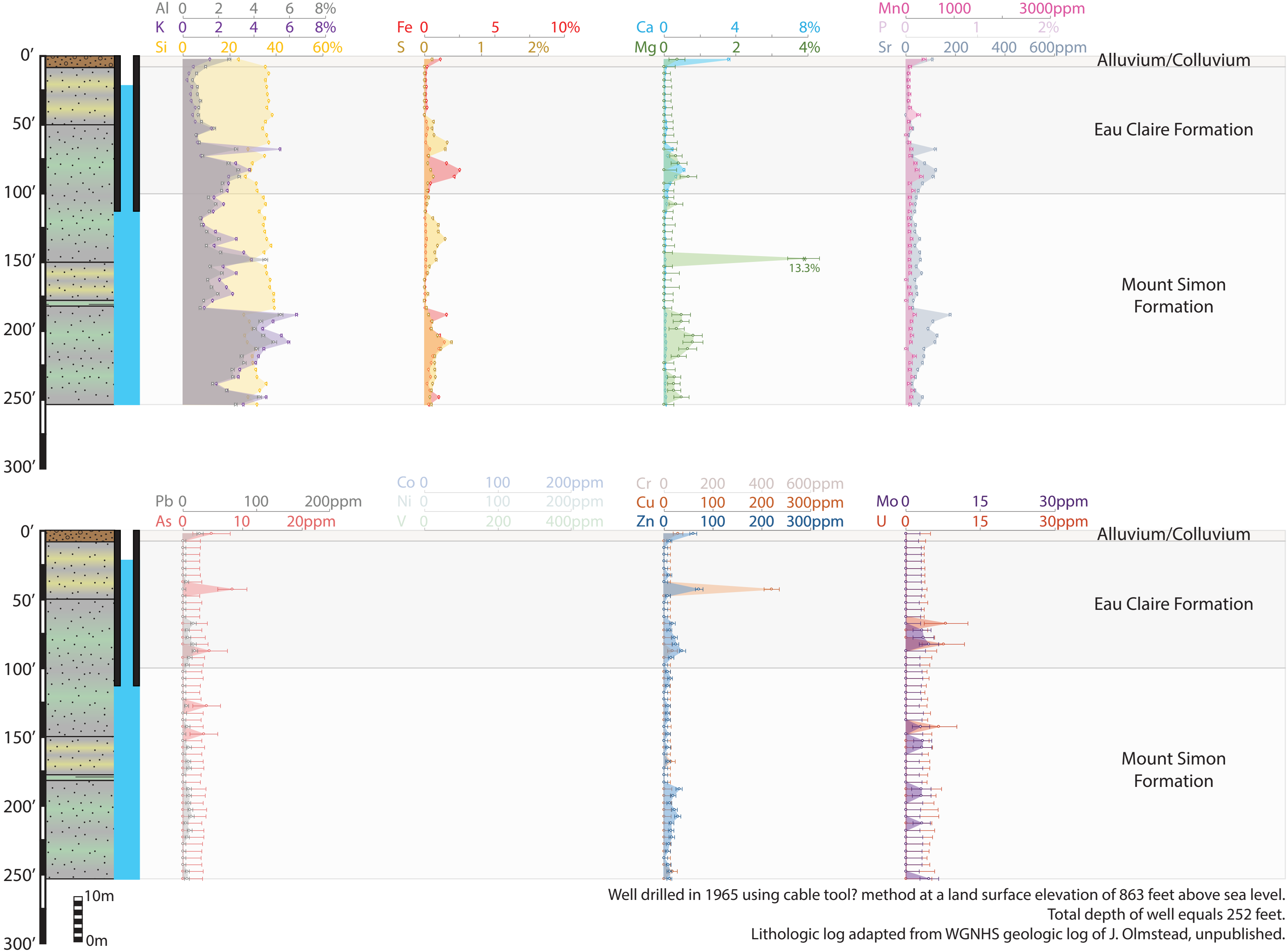

Well drilled in 1965 using cable tool? method at a land surface elevation of 863 feet above sea level.
Total depth of well equals 252 feet.
Lithologic log adapted from WGNHS geologic log of J. Olmstead, unpublished.

Well 13 - Blair City Well #5 (62000068)

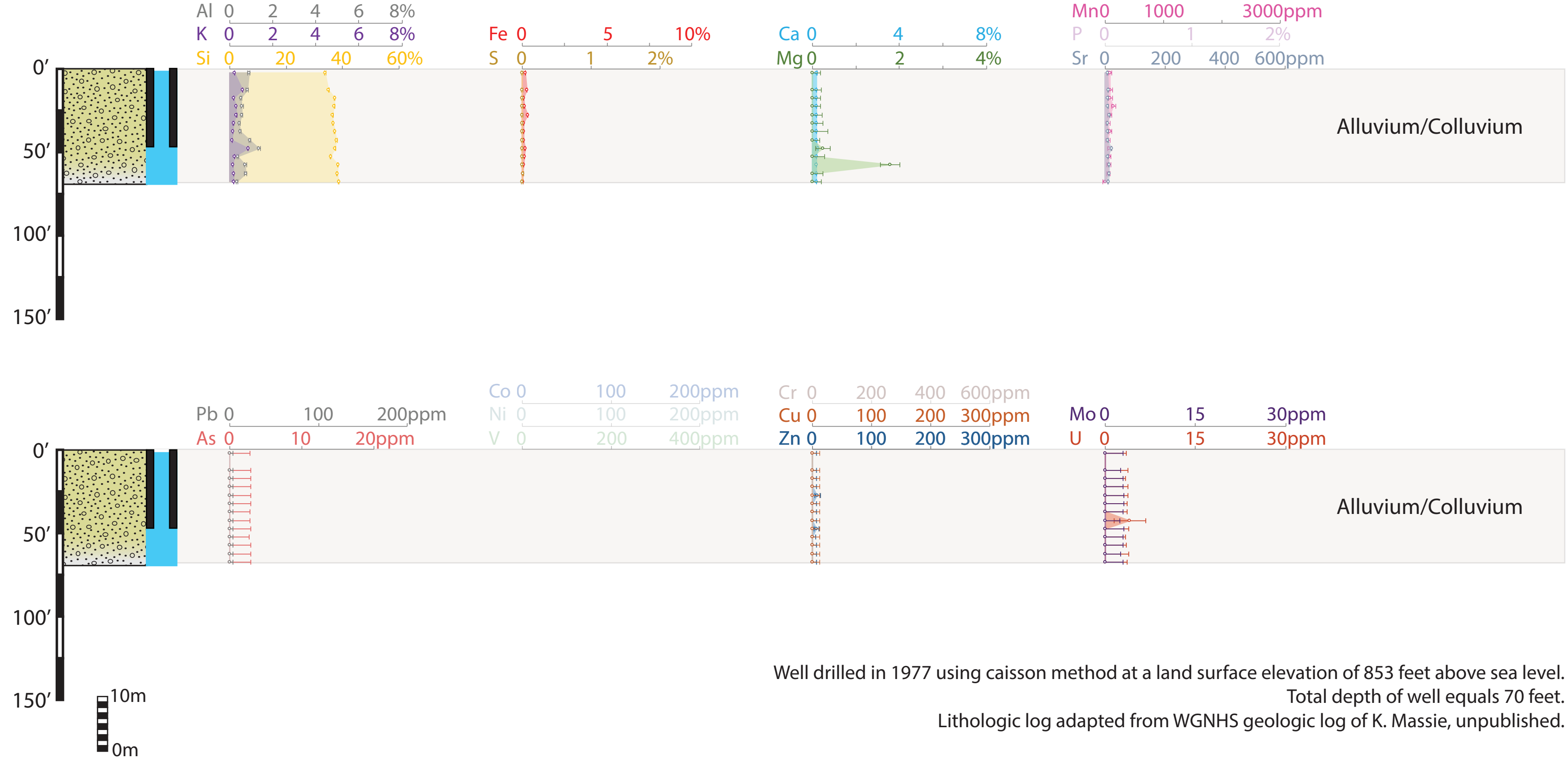

Well drilled in 1977 using caisson method at a land surface elevation of 853 feet above sea level.
Total depth of well equals 70 feet.
Lithologic log adapted from WGNHS geologic log of K. Massie, unpublished.

Well 14 - Blair City Test Hole #1-76 (62000070)

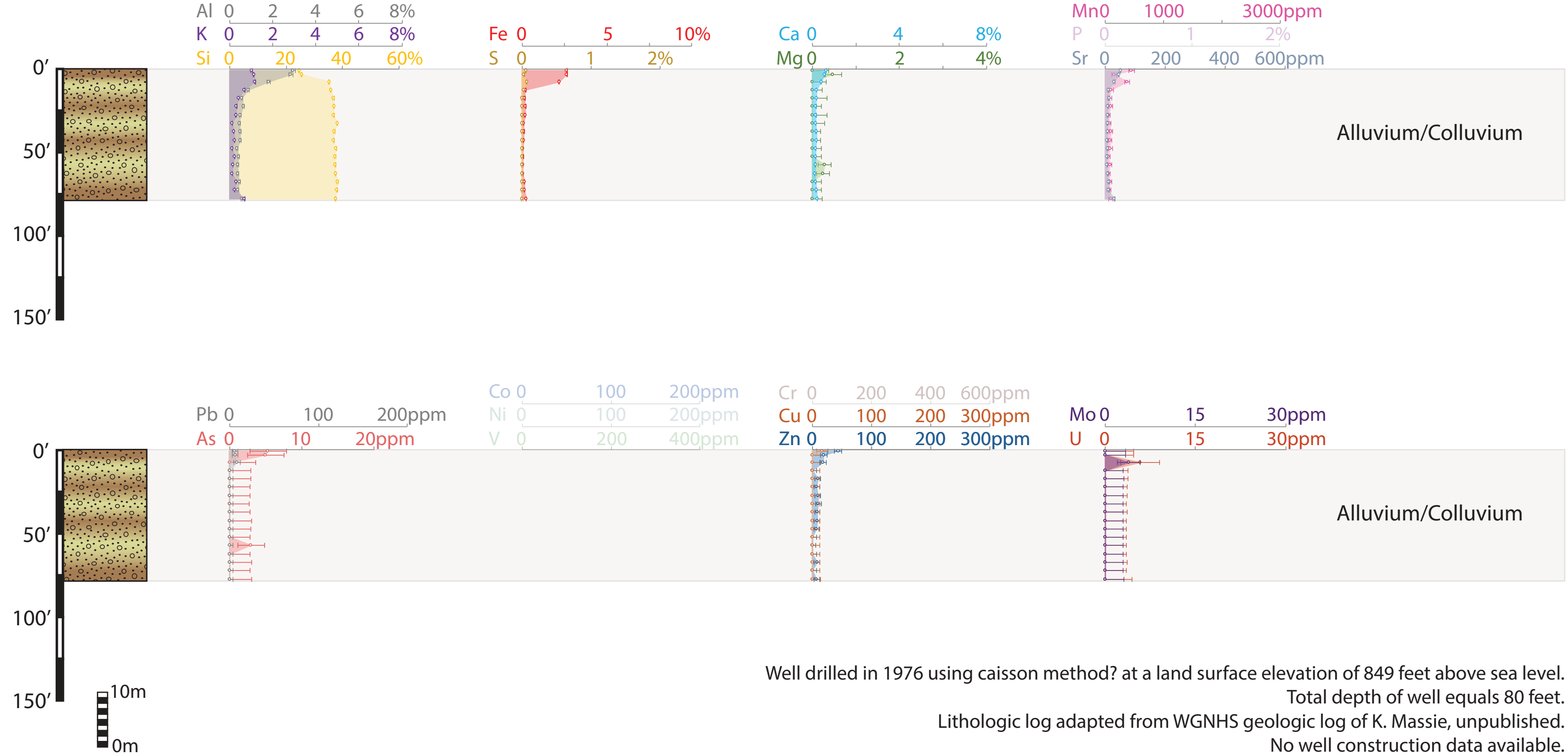

Well drilled in 1976 using caisson method? at a land surface elevation of 849 feet above sea level.
Total depth of well equals 80 feet.
Lithologic log adapted from WGNHS geologic log of K. Massie, unpublished.
No well construction data available.

Well 15 - Blair City Well #6 (62000109)

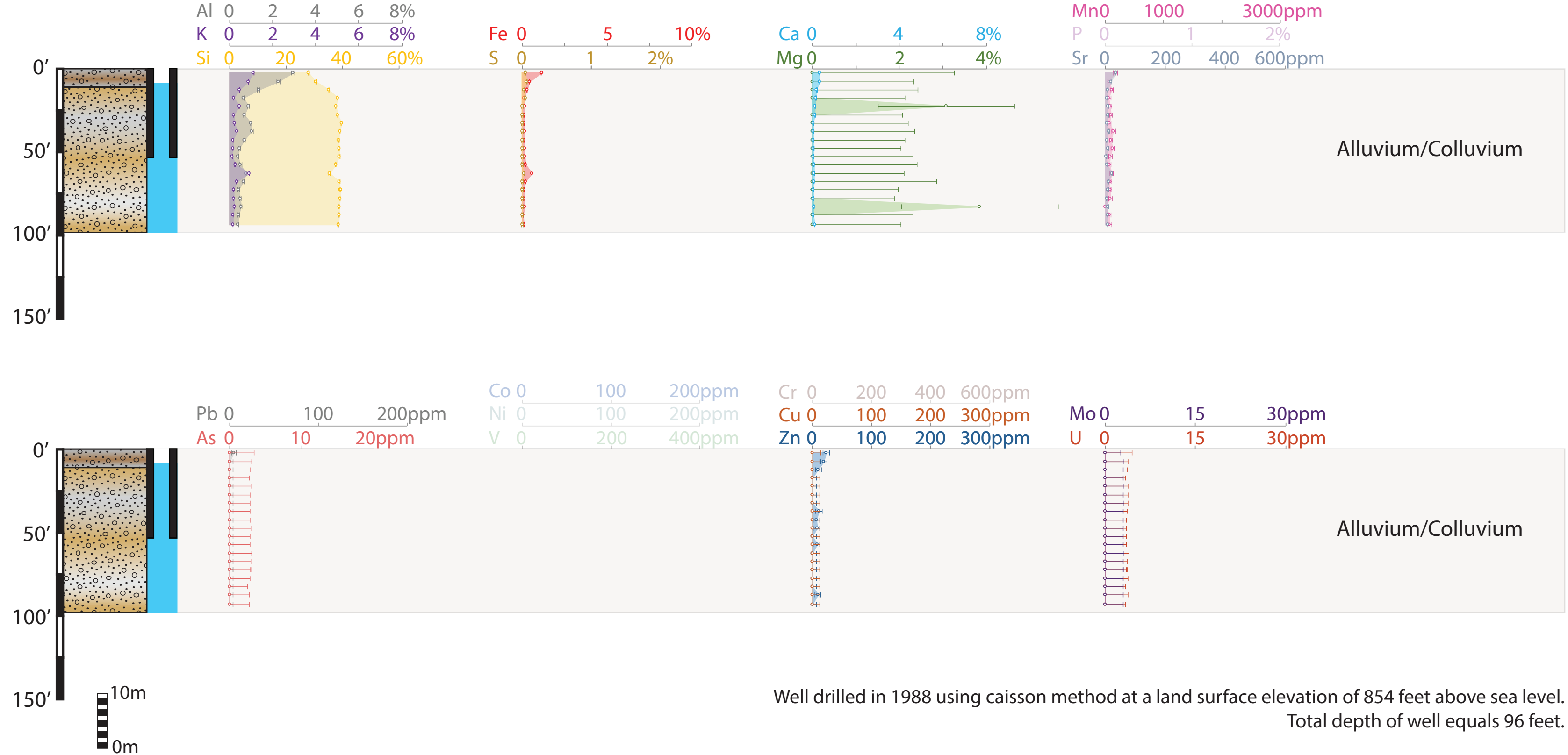

Well drilled in 1988 using caisson method at a land surface elevation of 854 feet above sea level.
Total depth of well equals 96 feet.

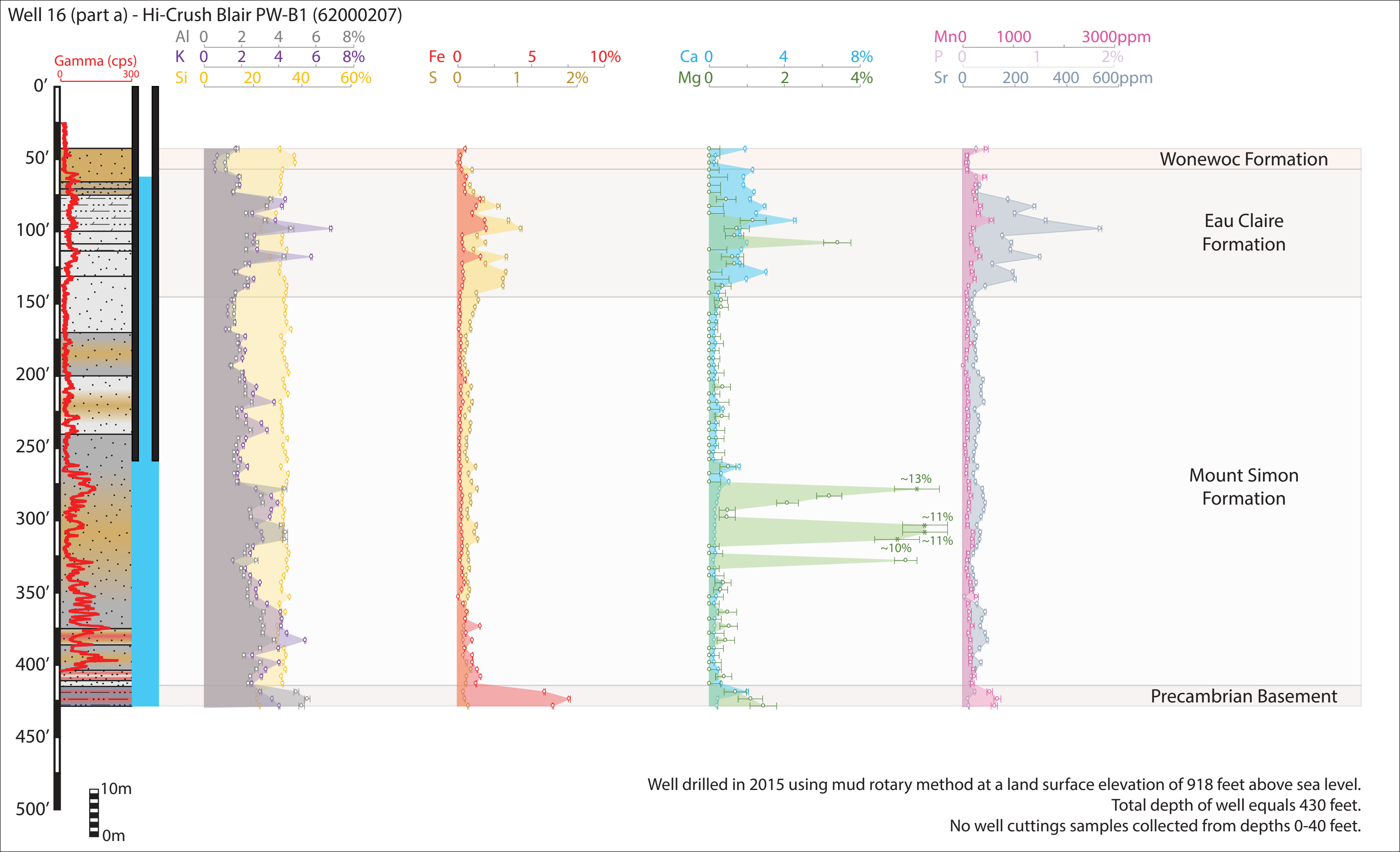

Well drilled in 2015 using mud rotary method at a land surface elevation of 918 feet above sea level.
Total depth of well equals 430 feet.
No well cuttings samples collected from depths 0-40 feet.

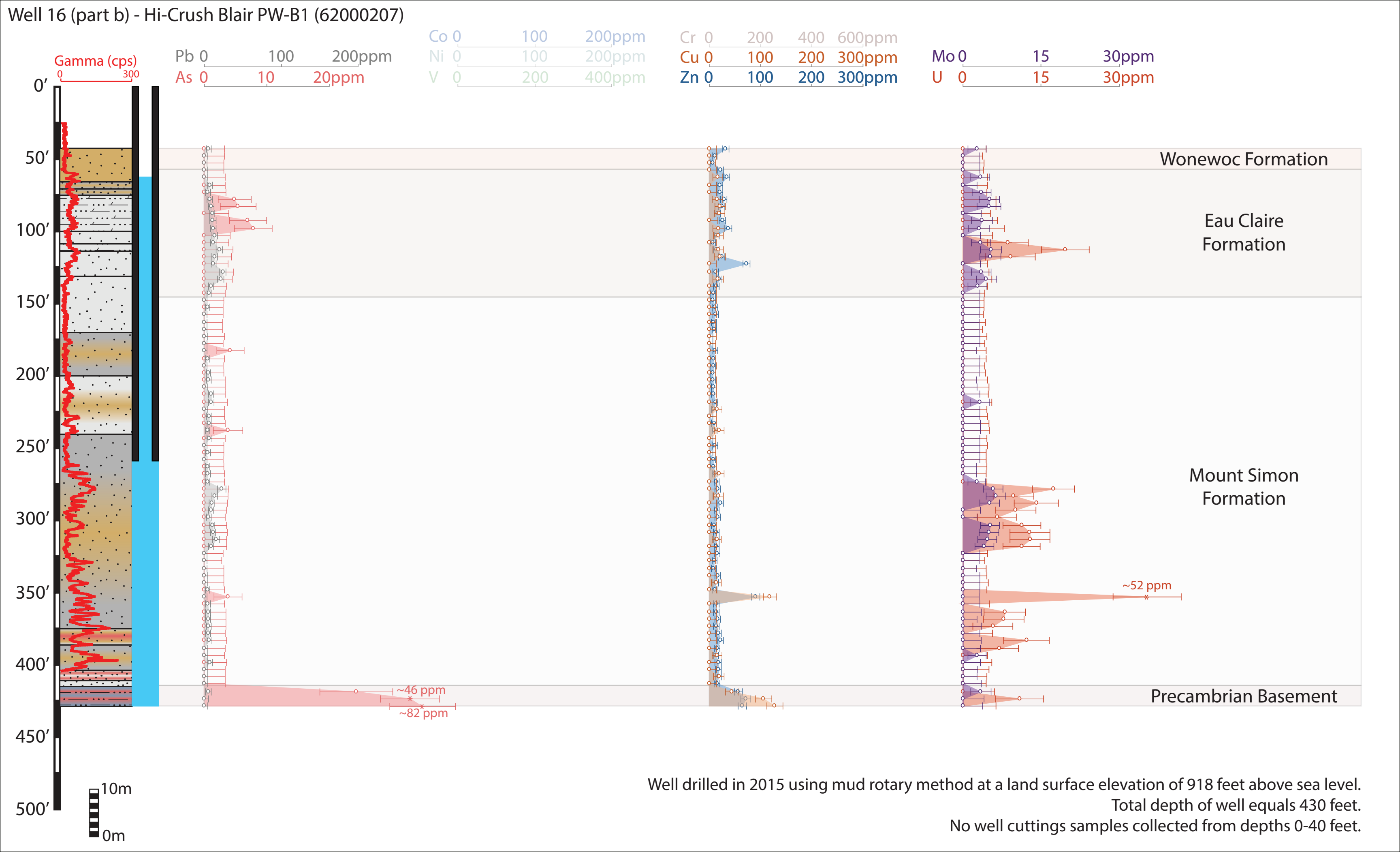

Well 16 (part b) - Hi-Crush Blair PW-B1 (62000207)
Gamma (cps)
0
300
Pb 0 100 200ppm
As 0 10 20ppm
Co 0 100 200ppm
Ni 0 100 200ppm
V 0 200 400ppm
Cr 0 200 400 600ppm
Cu 0 100 200 300ppm
Zn 0 100 200 300ppm
Mo 0 15 30ppm
U 0 15 30ppm
0'
50'
100'
150'
200'
250'
300'
350'
400'
450'
500'
10m
0m
Wonewoc Formation
Eau Claire Formation
Mount Simon Formation
Precambrian Basement
~46 ppm
~82 ppm
~52 ppm
Well drilled in 2015 using mud rotary method at a land surface elevation of 918 feet above sea level.
Total depth of well equals 430 feet.
No well cuttings samples collected from depths 0-40 feet.

Well 17 (part a) - Hi-Crush Blair PW-B2 (62000208)

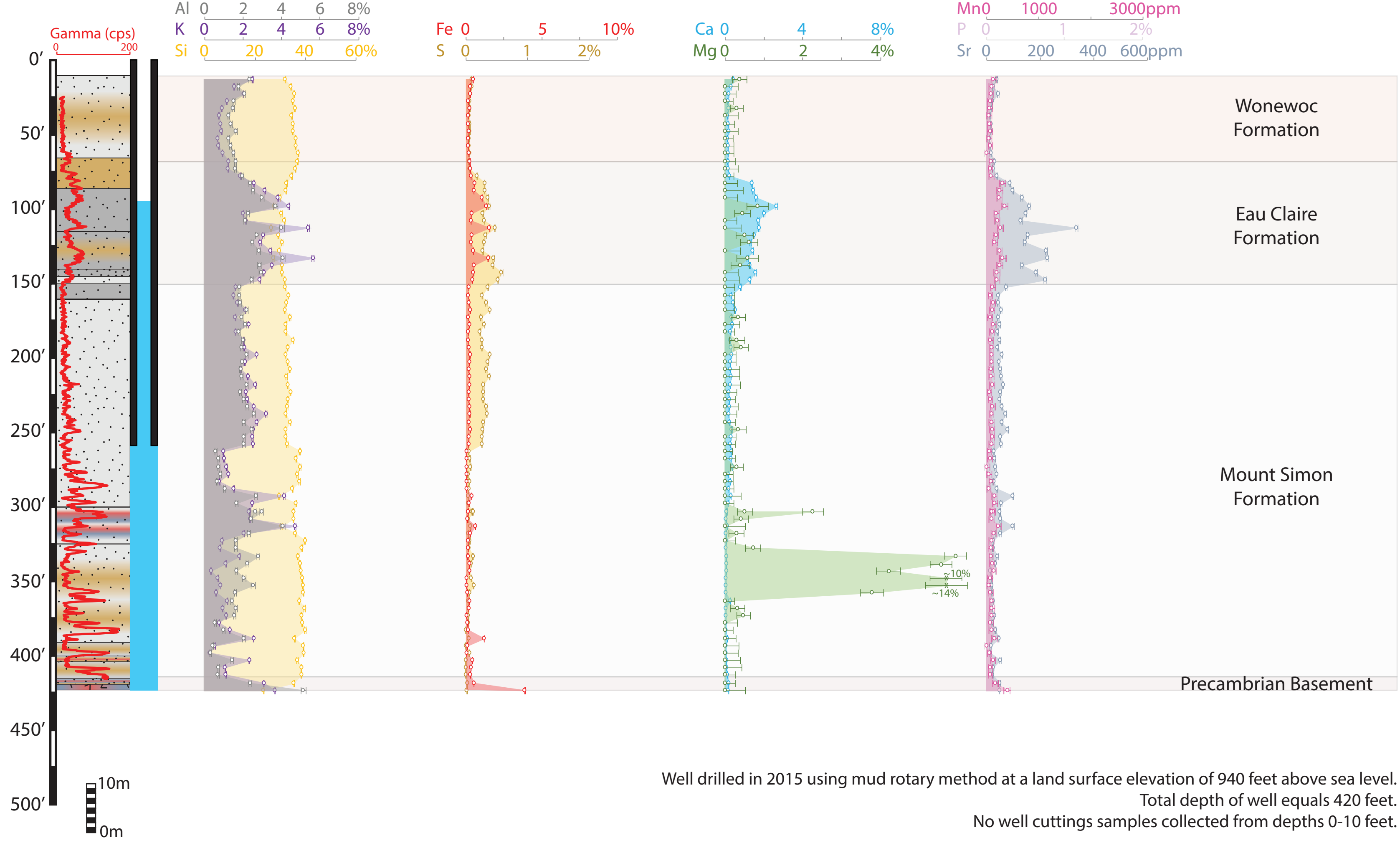

Well drilled in 2015 using mud rotary method at a land surface elevation of 940 feet above sea level.
Total depth of well equals 420 feet.
No well cuttings samples collected from depths 0-10 feet.

Well 17 (part b) - Hi-Crush Blair PW-B2 (62000208)

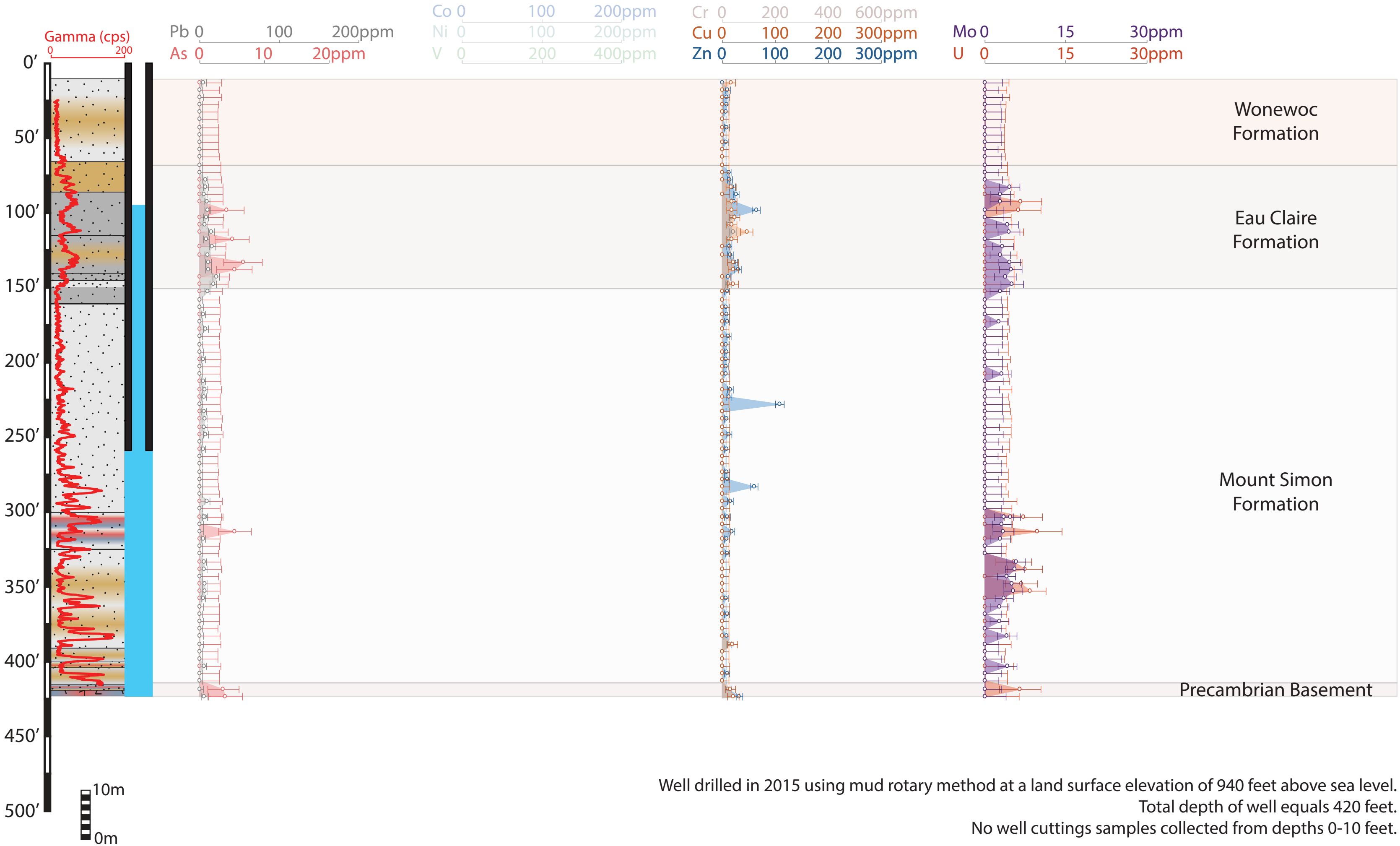

Well drilled in 2015 using mud rotary method at a land surface elevation of 940 feet above sea level.
Total depth of well equals 420 feet.
No well cuttings samples collected from depths 0-10 feet.

Well 18 (part a) - Hi-Crush Blair PW-B4 (62000209)

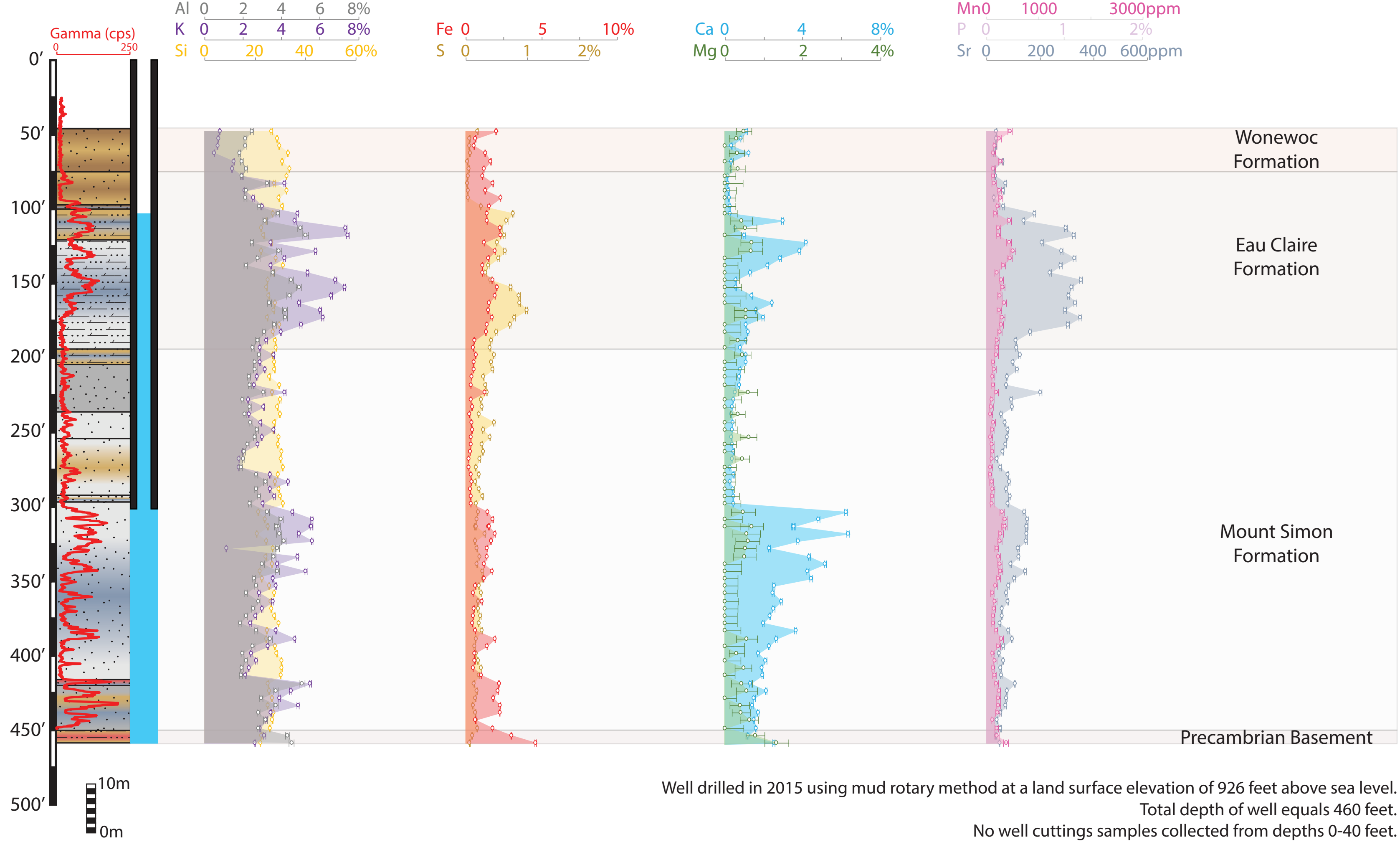

Well drilled in 2015 using mud rotary method at a land surface elevation of 926 feet above sea level.
Total depth of well equals 460 feet.
No well cuttings samples collected from depths 0-40 feet.

Well 18 (part b) - Hi-Crush Blair PW-B4 (62000209)

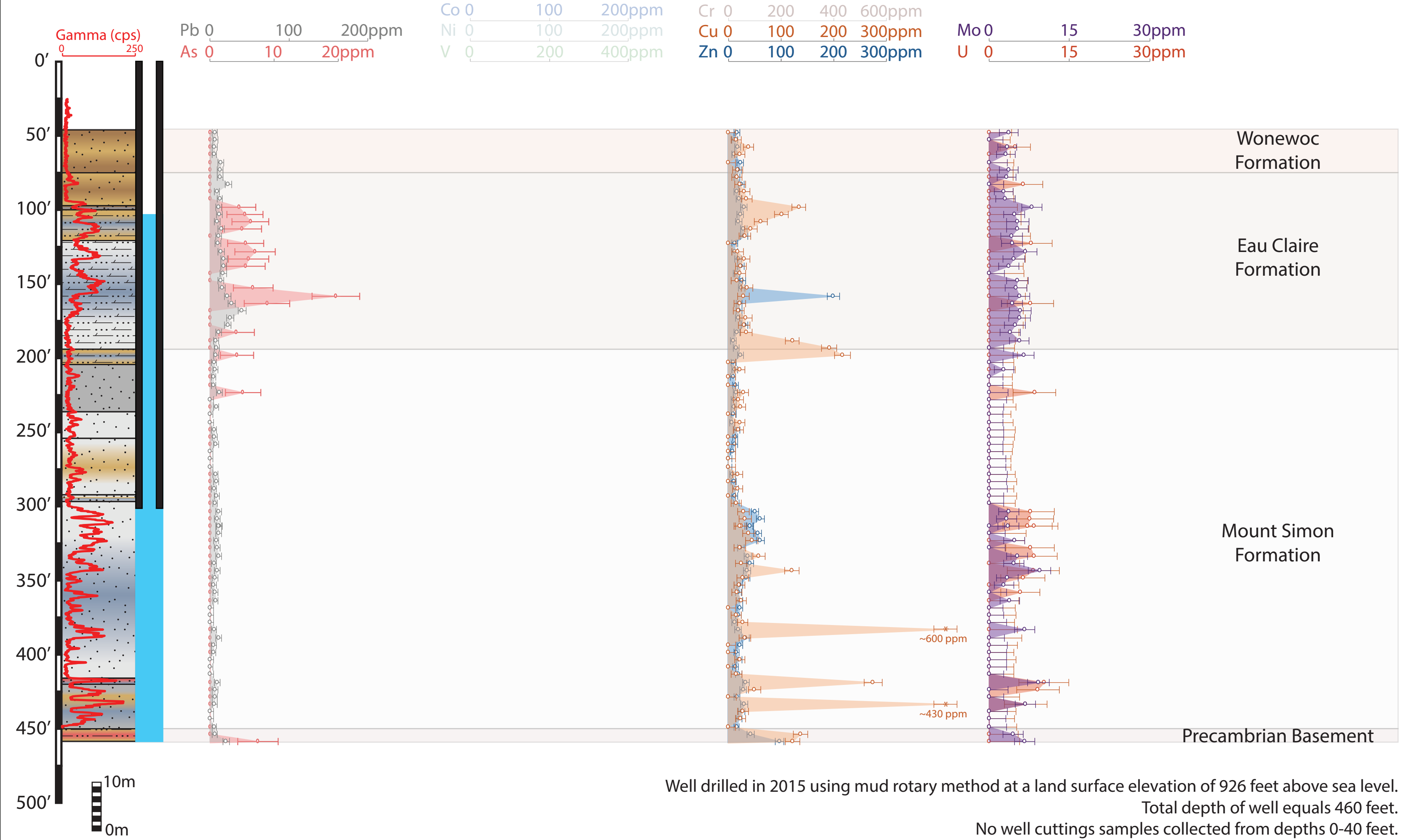

Well drilled in 2015 using mud rotary method at a land surface elevation of 926 feet above sea level.
Total depth of well equals 460 feet.
No well cuttings samples collected from depths 0-40 feet.

Well 19 (part a) - Hi-Crush Blair PW-B5 (62000210)

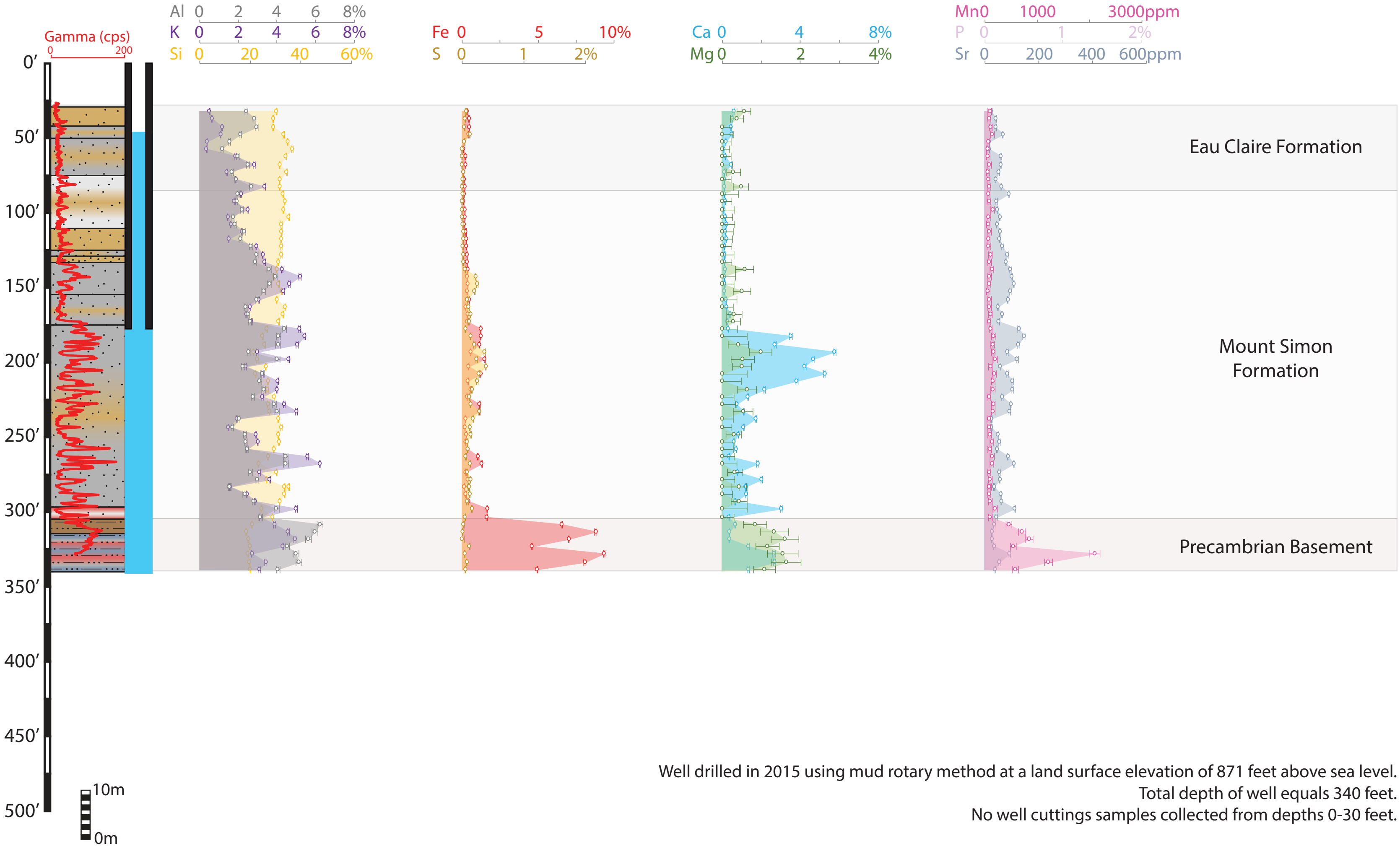

Well drilled in 2015 using mud rotary method at a land surface elevation of 871 feet above sea level.
Total depth of well equals 340 feet.
No well cuttings samples collected from depths 0-30 feet.

Well 19 (part b) - Hi-Crush Blair PW-B5 (62000210)

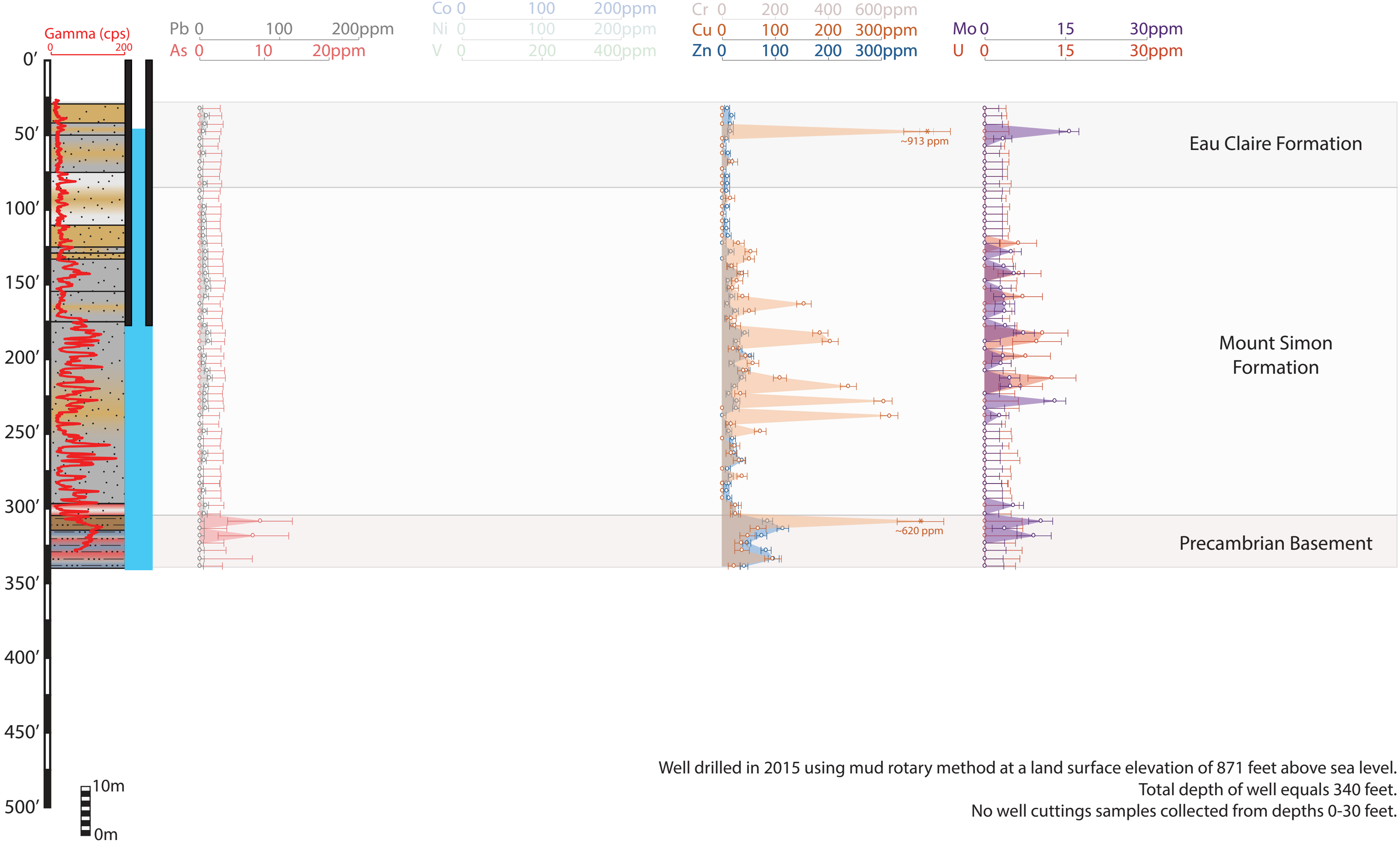

Well drilled in 2015 using mud rotary method at a land surface elevation of 871 feet above sea level.
Total depth of well equals 340 feet.
No well cuttings samples collected from depths 0-30 feet.

Well 20 (part a) - Hi-Crush Blair PW-B6 (62000211)

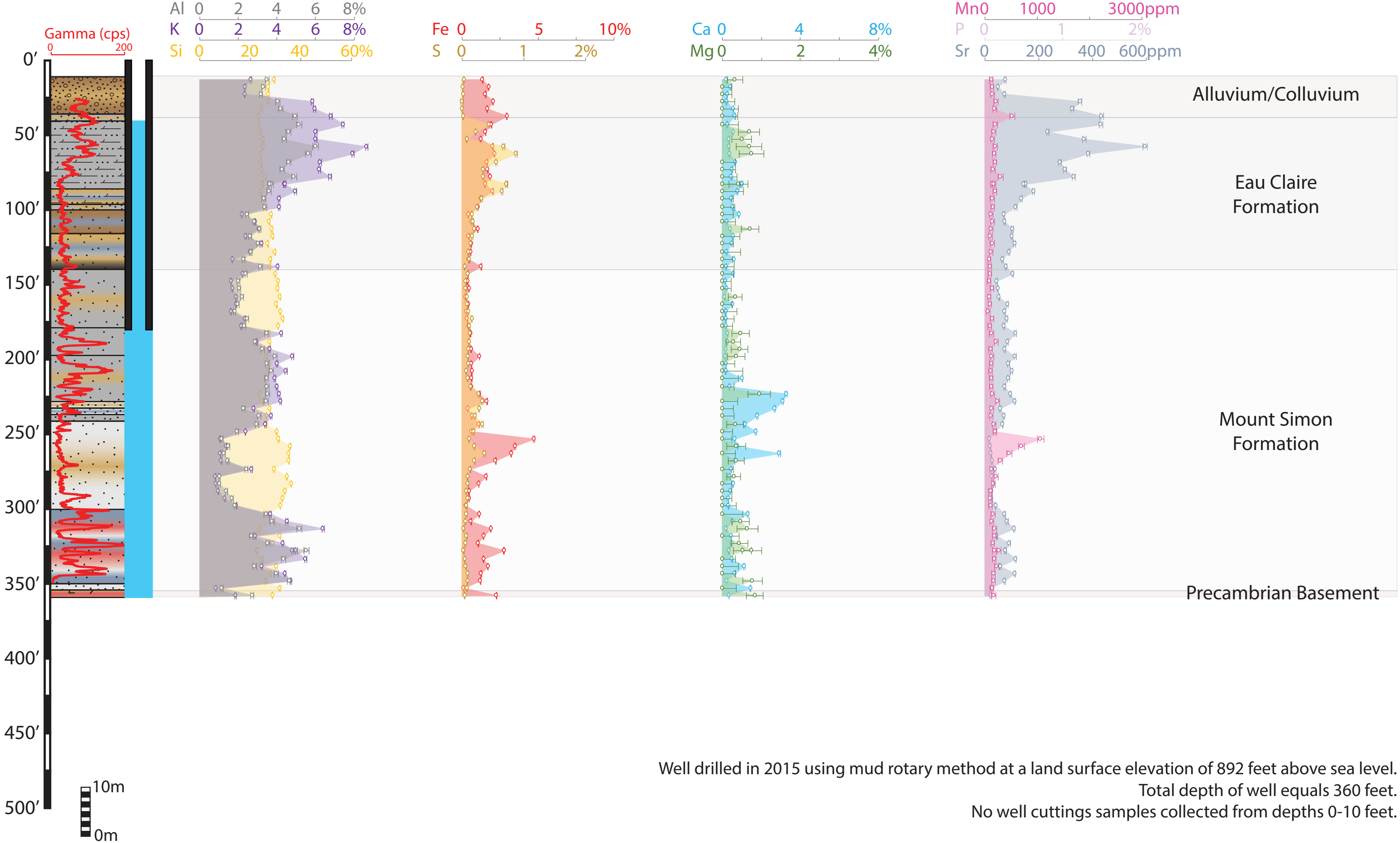

Well drilled in 2015 using mud rotary method at a land surface elevation of 892 feet above sea level.
Total depth of well equals 360 feet.
No well cuttings samples collected from depths 0-10 feet.

Well 20 (part b) - Hi-Crush Blair PW-B6 (62000211)

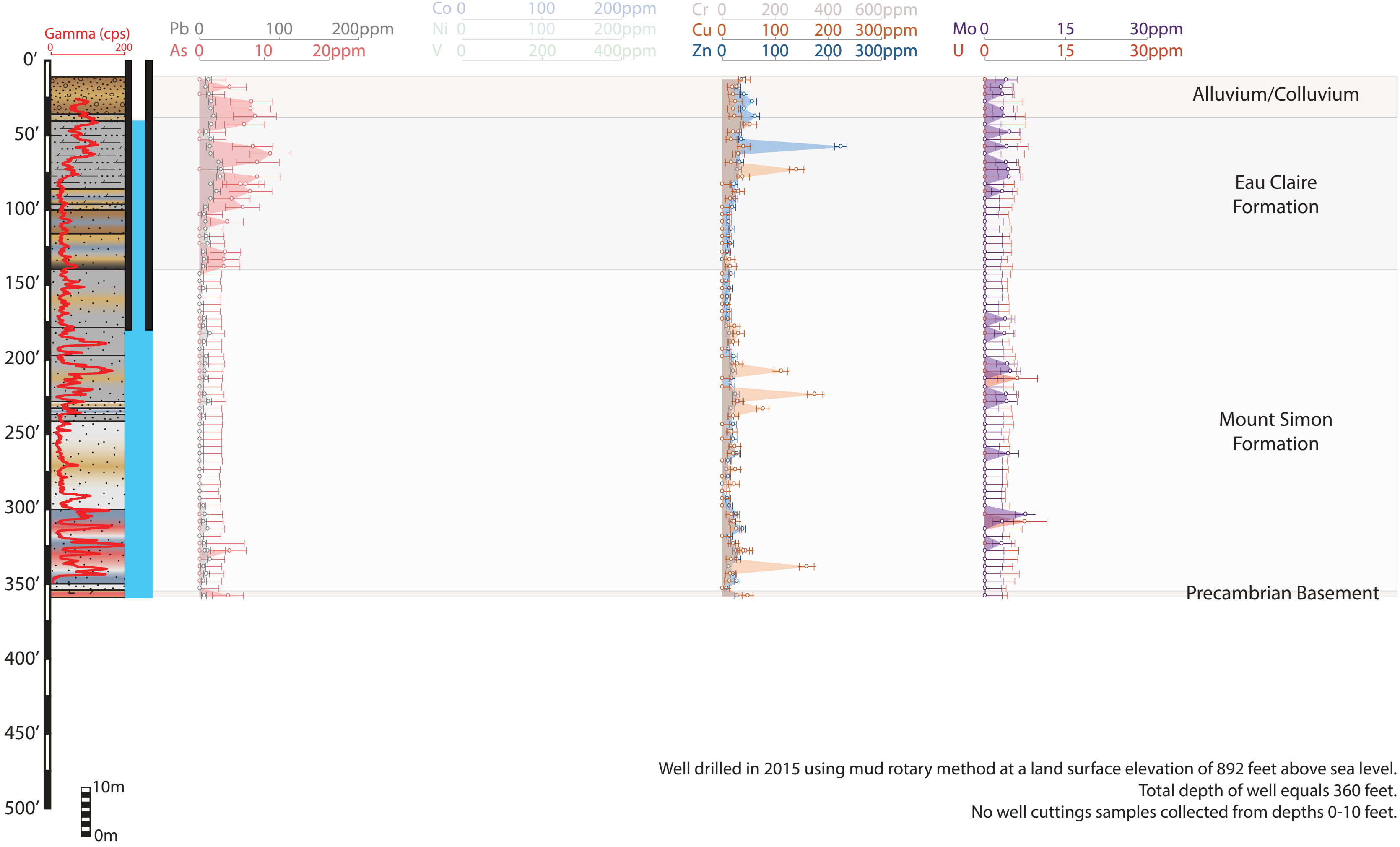

Well drilled in 2015 using mud rotary method at a land surface elevation of 892 feet above sea level.
Total depth of well equals 360 feet.
No well cuttings samples collected from depths 0-10 feet.

Well 21 - Kulig Quarry (62000231)

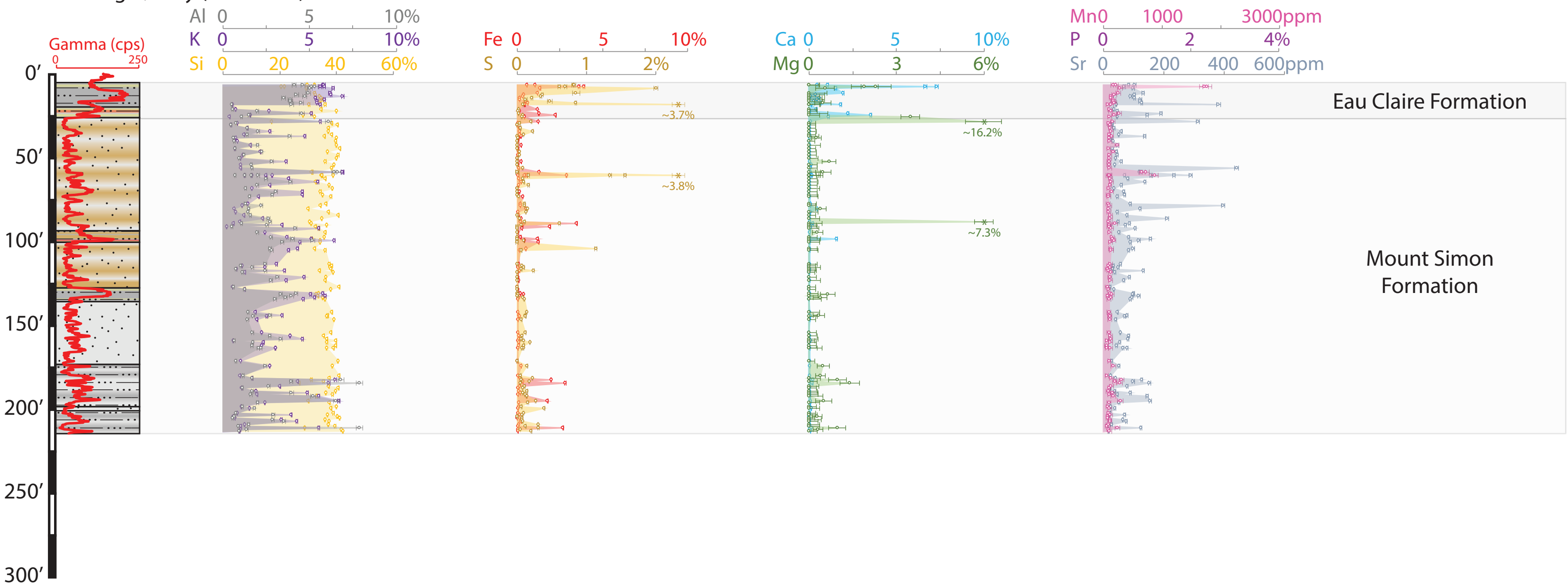

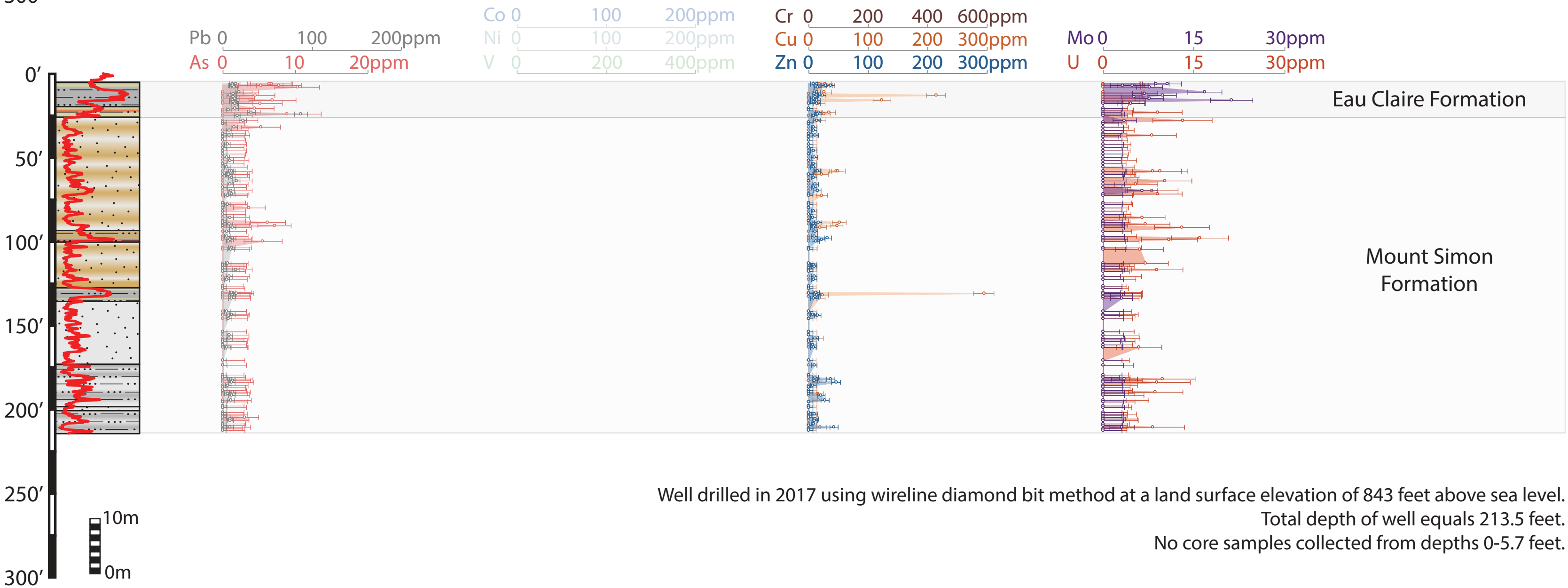

Well drilled in 2017 using wireline diamond bit method at a land surface elevation of 843 feet above sea level.
Total depth of well equals 213.5 feet.
No core samples collected from depths 0-5.7 feet.

Well 22 (part a) - Flambeau Mining Company 96-1-1 (62000119)

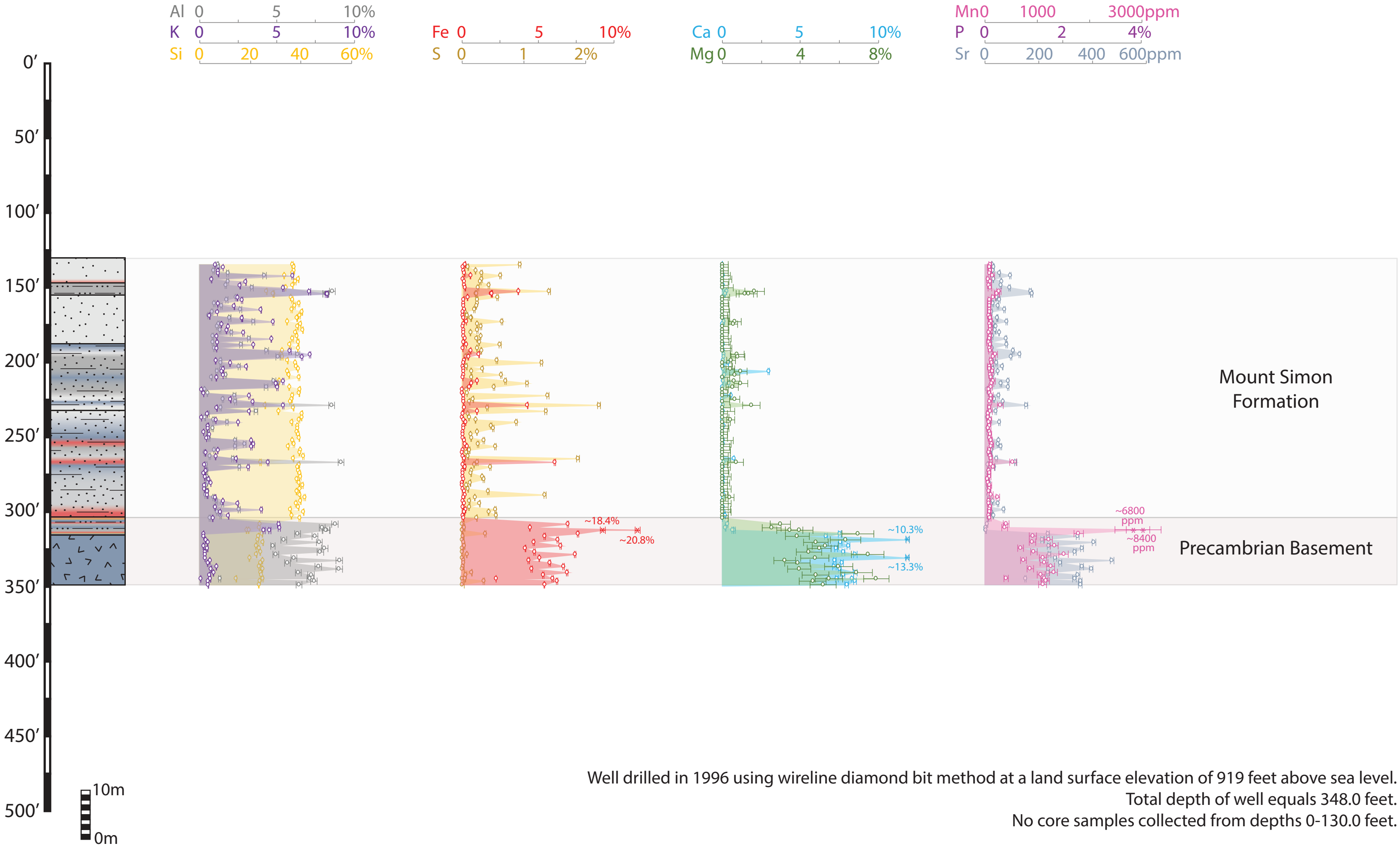

Well drilled in 1996 using wireline diamond bit method at a land surface elevation of 919 feet above sea level.
Total depth of well equals 348.0 feet.
No core samples collected from depths 0-130.0 feet.

Well 22 (part b) - Flambeau Mining Company 96-1-1 (62000119)

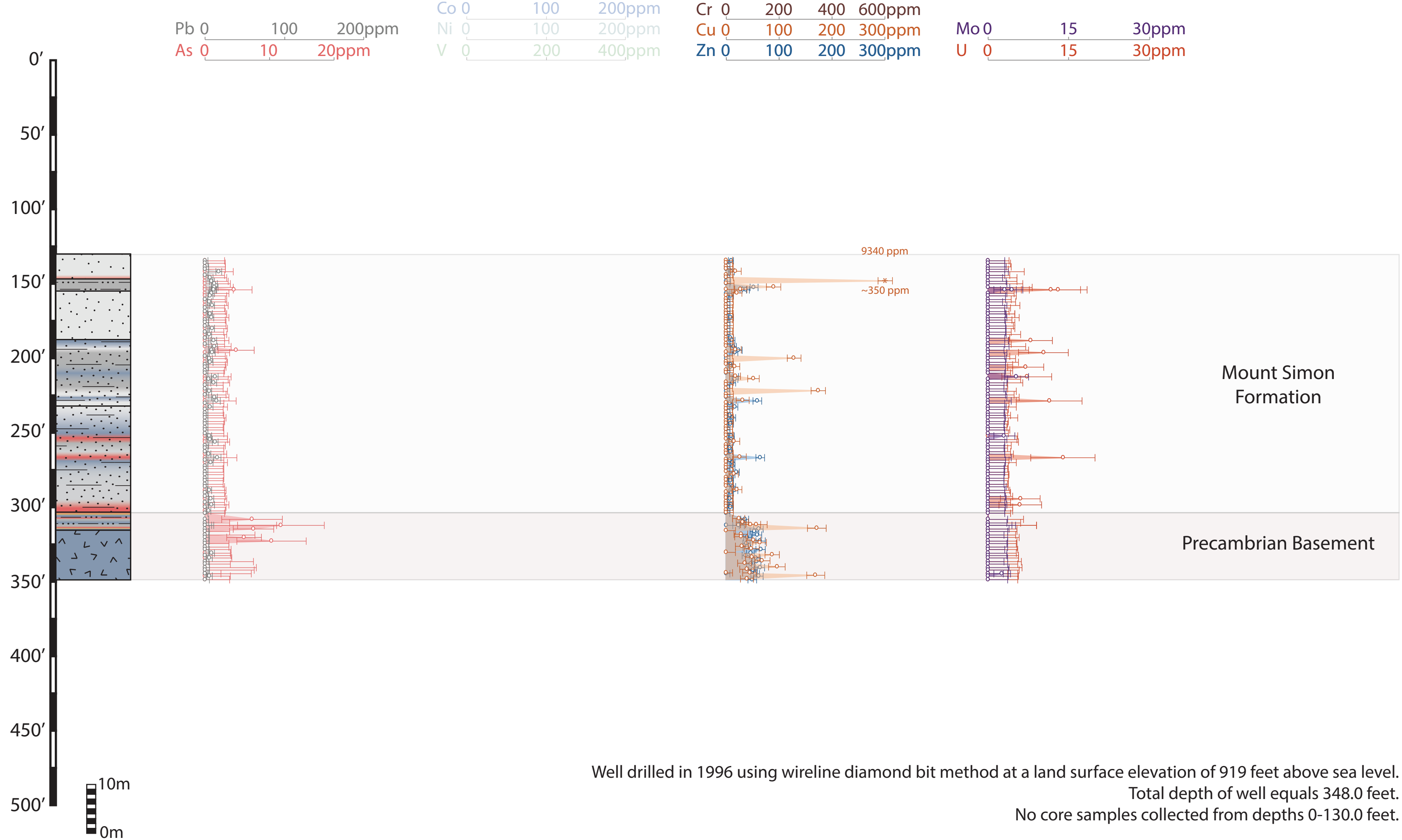

Well drilled in 1996 using wireline diamond bit method at a land surface elevation of 919 feet above sea level.
Total depth of well equals 348.0 feet.
No core samples collected from depths 0-130.0 feet.
