# Supplementary material for: A portable x-ray fluorescence (pXRF) elemental dataset collected from Cambrian-age sandstone aquifer material, Wisconsin, U.S.A
Source: Data Brief. 2022 Jun 23;43:108411. doi: 10.1016/j.dib.2022.108411 (PMC9251326; doi:10.1016/j.dib.2022.108411)

A portable x-ray fluorescence (pXRF) elemental dataset collected from Cambrian-age sandstone aquifer material, Wisconsin, U.S.A.

James J. Zambito IV, Lisa D. Haas, and Michael J. Parsen

zambitoj@beloit.edu

Appendix C

This appendix contains the available WGNHS geologic logs and downhole geophysical logs for boreholes studied.

Well #	Well Name	Year Drilled	WGNHS Geologic Log Available	Downhole Geophysical Log Available
1	Independence City Test Hole for Well #2 (62000114)	1997	NO	NO
2	Independence City Well #3 (62000137)	2009	NO	NO
3	Trempealeau County Hospital Well #5 (62000028)	1957	YES	NO
4	Land O'Lakes Creamery Well (62000024)	1953	YES	NO
5	Whitehall Test Hole #2 (62000018)	1953	YES	NO
6	Whitehall City Well #2 (62000023)	1953	YES	NO
7	Whitehall City Well (62000022)	1926	YES	NO
8	Whitehall City Sewage Treatment Plant Well (62000065)	1974	YES	NO
9	Winn Bay (Preferred) Sand Well (62000147)	2011	NO	NO
10	Blair City Well #3 (62000007)	1945	YES	NO
11	Associated Milk Producer, Inc. Well #3 (62000026)	1958	YES	NO
12	Blair City Well #4 (62000045)	1965	YES	NO
13	Blair City Well #5 (62000068)	1977	YES	NO
14	Blair City Test Hole #1-76 (62000070)	1976	YES	NO
15	Blair City Well #6 (62000109)	1988	NO	NO
16	Hi-Crush Blair PW-B1 (62000207)	2015	NO	YES
17	Hi-Crush Blair PW-B2 (62000208)	2015	NO	YES
18	Hi-Crush Blair PW-B4 (62000209)	2015	NO	YES
19	Hi-Crush Blair PW-B5 (62000210)	2015	NO	YES
20	Hi-Crush Blair PW-B6 (62000211)	2015	NO	YES
21	Kulig Quarry core (62000231)	2017	NO	YES
22	Flambeau Mining Co. 96-1-1 core (62000119)	1996?	NO	NO

TREMPEALEAU COUNTY HOSPITAL WELL #5

C, S½, NE¼, NE¼, NW¼, SE¼, sec. 21, T. 22 N., R. 8 W.
E. C. Finch & Son, Drillers, 4/6/57
Samples examined by F. T. Thwaites, Nos. 194935-194975

Elevation = 880'

Formation	Thickness	Depth	Thickness	Description	Well construction
SU	15	0-5	5	Sand, fine to medium, silty, brown-grey	16" pipe
		5-15	10	Sand, fine to medium, brown-yellow-gray	
EAU CLAIRE		15-20	5	Siltstone, sandy, light grey	
		20-35	15	Sandstone, very fine to coarse, silty, yel-gy	
		35-45	10	Siltstone, sandy, yellow-gray	40
		45-55	10	Sandstone, very fine to medium, silty, lt. yellow-gray	15" hole
		55-80	25	Sandstone, fine to coarse, light yellow-gray	10" pipe cemented
		80-105	25	Sandstone, very fine to coarse, light yellow-gray	80 water; 90; 99
		105-ℝ29	15	Sandstone, very fine to coarse, lt. yellow-gray	
		120-130	10	Sandstone, very fine to medium, lt. yel.-gy	10" hole
		130-160	30	Sandstone, very fine to medium, light gray	
		160-175	15	Sandstone, very fine to coarse, lt. yelllow-gray	
		175-185	10	Sandstone, very fine to fine, light gray	
		185-190	5	Sandstone, very fine to coarse, silty, light g	
	195	190-210	20	Siltstone, sandy, light gray	

Formations: Surface; Eau Claire
Tested 5 hours at 195 g.p.m. specific capacity = 3.6 g.p.m./ft.
Additional copies may be secured from Wisconsin Geological Survey, Science Hall, Madison 6, Wis.

LAND O' LAKES CREAMERY WELL NO. 2, WHITEHALL, WIS.
W. end of town Sec. 23, T. 22 N., R. 8 W.
Keys Well Drilling Co., 1953
Samples examined by F. T. Thwaites, Wisconsin Geological Survey,
Nos. 164531-164573

Formation	Thickness	Depth	Feet	Description	Well construction
SURFACE		0-10	10	Sand, medium to fine, light yellow-gray	
		10-15	5	Sand, fine to coarse, light yellow-gray	
		15-20	5	Sand, medium to fine, light yellow-gray	15.5 water
		20-25	5	Sand, fine to medium, light yellow-gray	
	50	25-50	25	Sand, fine to coarse, light yellow-gray; some glauconite (soft sandstone)	24" pipe
MT SIMON		50-75	25	Sandstone, fine to coarse, light gray, some glauconite	
		75-115	40	Sandstone, coarse to fine, light gray	107'2"
		115-120	5	Sandstone, fine to coarse, light gray	
		120-125	5	Sandstone, fine to medium, light gray	
		125-130	5	Shale, silty, green-gray	
		130-140	10	Sandstone, fine to medium, silty, light gray	23" hole
		140-150	10	Sandstone, very fine, silty, light gray	
		150-160	10	Siltstone, gray	
		160-165	5	Sandstone, silty to medium, light gray	
		165-170	5	Sandstone, fine to medium, silty, light gray	
	164	170-210	40	Sandstone, fine to coarse, silty, light gray	
		210-214	4	Shale, red,	

Tested 10 hours at 500 g.p.m. specific capacity = 4.25 g.p.m./ft.
Additional copies may be secured from Wisconsin Geological Survey, Sicence Hall, Madison 6, Wis.

TEST WELL NO. 2, WHITEHALL, WIS.

Sec. 23, T. 22 N., R. 8 W.
Egerer-Galloway Well Corporation, Contractors, 1953
G a rnock Engineering Co., Eau Claire
Samples examined by F. T. Thwaites, Nos. 163110-163147

Formation	Depth	Thickness	Description
	0-10	10	Sand, silty to medium, light brown-gray
	10-25	15	Sandstone, fine to coarse, light brown-gray
	25-40	15	Sandstone, fine to medium, light yellow-gray
EAU CLAIRE	40-50	10	Sandstone, coarse to fine, light yellow-gray
	50-55	5	Sandstone, fine to coarse, yellow-gray
	55-60	5	Sandstone, fine to medium, light gray
	60-65	5	No sample
	65-70	5	Sandstone, coarse to medium, light gray
	70-75	5	Sandstone, fine to medium, light gray
	75-85	10	Sandstone, medium to fine, light gray
	85-95	10	Sandstone, fine to medium, light gray
	95-110	15	Sandstone, fine to coarse, light gray
	110-120	10	Sandstone, fine to silty, light gray
	120-125	5	Sandstone, fine to medium, light gray
	125-130	5	Sandstone, fine to coarse, light gray
	130-145	15	Sandstone, fine to medium, light gray
MT SIM	145-150	5	Sandstone, very fine, light gray
	150-160	10	Sandstone, fine to coarse, light gray
	160-165	5	Sandstone, fine to very fine, light gray
	165-169	4	Sandstone, fine to medium, light gray
	169-174	5	Shale, sandy, red
	174-180	6	Sandstone, silty to very fine, light gray
	180-190	10	Sandstone, fine to medium, light gray

Formations: Eau Claire and Mt. Simon undivided

CITY WELL NO. 2, WHITEHALL, WIS.
$NW\frac{1}{4}$, $NW\frac{1}{4}$, $NE\frac{1}{4}$, Sec. 26, T. 22 N., R. 8 W.
Egerer-Galloway Well Corporation, Contractors, 1953
Garnock Engineering Co., Engineers
Wisconsin Geological Survey — Samples examined by F. T. Thwaites, Nos. 164475-164530

Formation	Total	Depth	Thickness	Description	Remarks
SURF	35	0-15	15	Sand, fine to coarse, light yellow-gray	18_2 pipe
		15-25	10	Sand, fine to coarse, light gray	
		25-35	10	Sand, fine to coarse, dark yellow-brown	29 water
MT SIMON	237	35-55	20	Sandstone, coarse to fine, light gray, slight yellow tinge	12" W.I. pipe cemented
		55-85	30	Sandstone, coarse to fine, light gray	
		85-90	5	Sandstone, fine to coarse, light gray	90
		90-100	10	Sandstone, coarse to fine, light gray	
		100-105	5	Sandstone, fine to medium, light gray	
		105-110	5	Sandstone, coarse to fine, light gray	
		110-115	5	Sandstone, fine to coarse, light gray	
		115-135	20	Sandstone, coarse to fine, light gray	12" hole
		135-150	15	Sandstone, fine to medium, light gray	
		150-155	5	Sandstone, very fine to coarse, light gray	
		155-160	5	Siltstone, dark gray; sandstone, fine, gray	
		160-165	5	Sandstone, very fine to coarse, light gray	
		165-170	5	Sandstone, coarse to fine, light gray	
		170-175	5	Shale, silty, red; sandstone, fine to med, pk	
		175-185	10	Sandstone, coarse to fine, light gray	
		185-190	5	Sandstone, fine to coarse, light gray	
		190-195	5	Sandstone, coarse to fine, light gray	
		195-205	10	Sandstone, fine to coarse, light gray	
		205-210	5	Sandstone, coarse to fine, light gray	
		210-215	5	Sandstone, fine to coarse, light gray	
		215-230	15	Sandstone, coarse to fine, light gray	
		230-235	5	Sandstone, fine to coarse, light gray	
		235-240	5	Sandstone, fine to medium, light gray	
		240-265	25	Sandstone, fine to coarse, light gray	
		265-274	9	Sandstone, coarse to fine, light gray	
		274-275	1	Shale, pink, green-gray (top pre-Cambrian?)	

Formations: Surface (alluvial); Mt. Simon; possibly pre-Cambrian
Tested at 525 g.p.m. specific capacity = 6.2 g.p.m./ft.
Additional copies may be secured from Wisconsin Geological Survey, Science Hall, Madison 6, Wis.

CITY WELL, WHITEHALL, WIS.
B. I. Baley and Sons, Drillers, 1926
Samples examined by F. T. Thwaites, Nos.77706-77745

NW¼, NW¼, NE¼, sec. 26, T22N, R8W
Elevation = 815'

DRIFT	112	0-112	112		No record, probably sand and gravel
MT. SIMON	153	112-140	28		Sandstone, medium to coarse, gray
		140-150	10		Shale, gray, with sandstone like above
		150-160	10		Shale, gray
		160-180	20		Sandstone, exceedingly coarse, gray shale
		180-195	15		Sandstone, exceedingly coarse to fine, gray
		195-210	15		Sandstone, fine to medium, light gray
		210-245	35		Sandstone, very coarse to fine, light gray
		245-250	5		Ss., like above, hard, very fine yellow layers
		250-255	5		Ss., exceedingly coarse to medium, light gray
		255-265	10		Ss., like above, angular pink feldspars
PЄ	59	265-280	15		Granite, pink, with much caved sandstone
		280-285	5		Clay, gray, decomposed rock
		285-310	20		Clay, red and gray=decomposed rock
		310-315	5		Gabbro and granite, decomposed
		315-324	9		Gabbro

Formations: Drift (outwash); Mt. Simon; pre-Cambrian

UNIVERSITY OF WISCONSIN GEOLOGICAL & NATURAL HISTORY SURVEY
1815 University Avenue, Madison, Wisconsin 53706

Log No F25-Tr-65

Well name Whitehall City Sewage Treatment Plant Well

County: Trempealeau

Owner..... City of Whitehall
Address.. City Hall
Whitehall, WI 54773
Driller.. Carl F. Schultz
Engineer.

Completed.... 11/30/74
Field check.
Altitude.... 835' ETM
Use......... Potable
Static w.l.. 29'
Spec. cap... 2.3 GPM/ft.

R. 8W.
T. 22 N.
Sec. 24

Quad. Whitehall 7½'

Drill Hole						Casing & Liner Pipe or Curbing							
Dia.	from	to	Dia.	from	to	Dia.	Wgt.& Kind	from	to	Dia.	Wgt.& Kind	from	to
12"	0	51'				12"	New Bk Steel Welded points 45#	0	51'				
6"	51	151'				6"	New Bk steel T & C 19.18#	+14"	125				

Grout: Kind	from	to
Sand and clay	0	7'
Pressure grouted cement	7'	51'

Samples from 0 to 151' Rec'd: 3/13/75 Studied by: R. M. Peters Issued: 10/4/83

Formations: Surface, Alluvium, Mt. Simon Sandstone.

Remarks: Well tested 8 hours at 30 GPM with 13 feet of drawdown. The interval 100'-105' is a buried soil. The interval 105'-120' is weathered bedrock over bedrock. Well drilled by cable tool method.

LOG OF WELL:

	Depths	Graphic Section	Rock Type	Color	Grain Size Mode	Grain Size Range	Miscellaneous Characteristics
Sfc.	0-5		Sand	Dk yl bn	M	Vfn/VC	Little soil. Trace glauconite, silt.
10'	5-10		"	"	"	"	Same.
A	10-15		St & Snd	Yl brown	"	"	Trace glauconite.
L	15-20		"	"	"	"	Little glauconite.
L	20-25		Sand	Bn yellow	"	"	Little silt. Trace glauconite, gravel, glauconitic sandstone.
U	25-30		"	"	"	"	Much silt. Little glauconite.
V	30-35		"	"	"	"	Little silt, glauconite.
I	35-40		"	"	"	"	Same plus little gravel (glaucic sandstone).
U	40-45		"	Pl yellow	"	"	Trace glauconite.
M	45-50		"	"	"	"	Little glauconite. Tr silt, gravel (glaucic sandstone).
	50-55		"	Lt yl bn	"	"	Trace glauconite, silt.
	55-60		"	"	"	"	Little organic matter (cvd?). Tr glauconite, st, granules.
	60-65		"	Gray bn	"	"	Little glauconite, silt.
	65-70		St & snd	Dk gy bn	"	"	Mch organic matter (caved?). Trace glauconite.
	70-75		Sand	Lt gray	"	"	Trace glauconite.
	75-80		"	Gry brown	"	"	Little silt. Trace glauconite.
	80-85		"	"	"	"	Much silt. Trace glauconite, organic matter (caved?).
	85-90		"	"	"	"	Same but little silt.
	90-95		Clay	V dk gy bn	--	--	Much sand, silt. Trace gravel, glauconite.
	95-100		Clay & Snd	"	M	Vfn/C	Much silt. Trace glauconite. Dolic&siliceous (clay).
	100-105		Silt	Black	--	--	Siliceous. Much clay, organic matter, sand. Tr glauconite.
	105-110		Sand	Lt yl bn	C	Vfn/VC	Ltl grvl (sil cemtd micaceous Vfn/fn sandstone). Tr glauconite.
	110-115		"	"	"	"	Same.
110'	115-120		"	Lt yl gy	"	"	Mch gvl (sil cemtd micac-foss Vfn/fn ss). Tr bn cl, bl&gn glauc.
Mt.	120-125		Sandstone	"	"	"	Wthrd. Disagg. Ltl sil cemtd micac fossilif ss. Tr Vfn/C glauc.
S.	125-130		"	White	M	Vfn/C	Tr Vfn/fn ss as in 120-125', Vfn/fn glauc, gy cl, sil cement.
	130-135		"	"	M/C	Vfn/VC	Tr Vfn/fn ss as in 120-125', Fn glauc, bn cl, sil&pyr cem, gy sh.
	135-140		"	"	C	"	Trace silica cement, pyrite cement, green gray shale.
	140-145		"	Lt yl gy	C/VC	"	Mny grans. Little green gray micaceous shale. Trace sil cem.
31'	145-151		"	"	C	"	Ltl green gray micac shale. Tr pyrite&silica cement, granules.
			END	OF LOG			

VILLAGE WELL NO. 3 , BLAIR, WIS.
S. P. Hall, Engineer
E. J. Fisher Well Drilling Co., Contractors, 1945
Samples examined by F. T. Thwaites, Nos. 125576-125613
NE$\frac{1}{4}$,SW$\frac{1}{4}$,NE$\frac{1}{4}$, Sec.16, T.21N., R.7W. Alt.= 860' ETM

Formation	Thickness	Depth	Ft.	Description	Construction
ALL		0-8	8	Silt, black	18" pipe
		8-13	5	Sand, very fine to silty, dark gray	19 water
	19'	13-19	6	Clay, silty, light gray	
E.C.		19-24	5	Shale, silty, light yellow-gray	20
		24-30	6	Shale, light gray	
	16'	30-35	5	Sandstone, fine to silty, light gray	
MT SIMON		35-40	5	Sandstone, medium to silty, light yellow-gy	
		40-45	5	Sandstone, medium to fine, light yellow-gy.	12" g.w.i. pipe cemented
		45-60	15	Sandstone, medium to fine, white	
		60-65	5	Sandstone, coarse to fine, light yellow-gy.	
		65-100	35	Sandstone, coarse to fine, light gray	86
		100-130	30	Sandstone, coarse to silty, light gray	12" hole
		130-140	10	Sandstone, medium to silty, light gray	
		140-155	15	Sandstone, coarse to silty, light gray	
		155-160	5	Sandstone, very fine, light gray	
		160-165	5	Sandstone, fine to medium, light gray	
		165-170	5	Sandstone, coarse to silty, light gray	
		170-180	10	Sandstone, medium to fine, light gray	
		180-185	5	Sandstone, medium to silty, light gray	
	160'	185-195	10	Shale, light gray	

Formations: Alluvium, Eau Claire Formation, Mt. Simon Sandstone.
Tested 24 hours at 300 g.p.m. specific capacity = 7.32 g.p.m./ft.

Sample log revised by R. M. Peters, 7/17/81.

WISCONSIN GEOLOGICAL SURVEY, Science Hall, Madison, Wisconsin — Log No. Tr-26

Now Western Wisconsin Dairies Cooperative

PRESTON CREAMERY WELL NO. 3, BLAIR, WISCONSIN
NW1/4, SE1/4 ~~East ½~~, Sec. 16, T 21N, R 7W, Gilbert Street
Layne-Northwest Co., Driller, October 1958
Sample Nos. 206439-206455 - Examined by J. B. Steuerwald

Formation		Depth (ft)	Thickness		Description
SURFACE		0 - 10	10		Soil, dark brown, sandy, little clay, few rounded igneous pebbles
		10 - 35	25		Sand, medium to very coarse, light gray
		35 - 50	15		Sand, medium & coarse, light gray
		50 - 85	35		Gravel, fine & medium, sandy, non dolomitic
	100	85 - 100	15		Samples missing

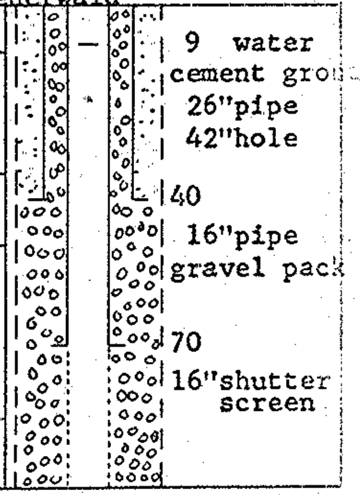

Tested for 8 hours @ 1300 gpm, specific capacity = 35 gpm/ft. of drawdown.

UNIVERSITY OF WISCONSIN GEOLOGICAL & NATURAL HISTORY SURVEY
1815 University Avenue, Madison, Wisconsin 53706

Log No. Tr-45
Issued: Nov., 1967

Well #4

County: Trempealeau

Well name City of Blair, Wis.
Located at Porter and Taft Sts.
Owner.... City of Blair, Wis.
Address.. c/o Clerk, City Hall
Blair, Wis.
Driller.. Keys Well Drilling Co.
Engineer.

Completed... 11/13/65
Field check.
Altitude.... 862' ETM
Use......... Municipal
Static w. l.= 19'11"
Spec. cap...= 5.3

R. 7W.
T. 21 N.
Sec. 16

Quad. Blair 15'

Drill Hole						Casing & Liner Pipe or Curbing							
Dia.	from	to	Dia.	from	to	Dia.	Wgt.& Kind	from	to	Dia.	Wgt.& Kind	from	to
19"	0	252'				20"	Blk steel 78#	0	104'				
						12"	Blk steel	+2'	114'				

Grout: Kind	from	to
Neat Cement	0	114'

Samples from 0 to 252 Date received: 11/17/65
Sample Nos. 259450 to 259500 Examined by: Janet Olmstead Date: 6/8/66
Formations: Surface, Mt. Simon,

Remarks: Well tested for 9 hours at 300 gpm with 56'11" of drawdown.

LOG OF WELL:

S	10'	0-5	5	G	Snd,pl bn,M&fn,Srnd,P srtg,mchVfn,ltlVC;ltl cl,st&fn gvl,trM,trglauc
		5-10	5	G	Snd,bnsh yl,M&C,rnd,P srtg,ltlVfn,mch fn;ltlFe stn,tr dol,glauc&cl
MT SIMON		10-20	10	G	Ss,bnsh yl,M&fn,Srnd,P srtg, ltl C; ltl Fe stn, tr glauc
		20-30	10	G	Ss, yl gry, M & fn, Srnd, P srtg, ltl C; tr glauc
		30-35	5	G	Ss, yl gry, M & C, rnd, P srtg, tr VC; tr cl & glauc
		35-50	15	G	Ss, yl gry, M & C, rnd, P srtg, ltl VC & fn, tr Vfn; tr cl & glauc
		50-55	5	G	Ss, lt ol gry, M & fn,rnd,P srtg,ltl C,tr Vfn;tr glauc & mica sh
		55-60	5	G	Ss, gn gry, M & C, rnd,P srtg, tr VC & Vfn, mch fn; tr glauc
		60-65	5	G	Ss, pl or, M & C, rnd,P srtg, ltl VC & fn,tr Vfn;tr glauc
		65-70	5	G	Ss,lt ol gry, fn & Vfn,Sang,F srtg,G Si-cem,trC&VC,ltlM;tr glauc&cht
		70-75	5	G	Ss,lt ol gry, M & C, rnd, P srtg, F Si-cem,mch fn,ltl Vfn;tr glauc
		75-80	5	G	Ss, ol gry,M & fn, Srnd,P srtg,slgt clacic,ltlC & Vfn;ltl ck,tr glauc
		80-85	5	G	Ss,ol gry,fn&Vfn,Sang,F srtg,slgt calcic,trM&C;ltl glauc&cl,tr mic
		85-90	5	G	Ss,gry bn,Vfn,Sang,P srtg,mch fn,ltlC,trM;mch cl&st,tr glauc
		90-95	5	G	Ss,lt ol gry,fn&Vfn,Sang,F srtg,G Si-cem,trM&C;tr glauc,ltl yl bn st
		95-100	5	G	Ss,lt ol gry,M&fn,Sang,P srtg,ltlC,trVC&Vfn;ltl glauc,tr st
		100-105	5	G	Ss,lt ol gry,fn&Vfn,Sang,F srtg,G Si-cem,trC&M;tr glauc
		105-110	5	G	Ss,Vlt ol gry,M&C,rnd,P srtg,F Si-cem,mchVC,ltl fn;tr glauc&cht
		110-120	10	G	Ss,Vlt ol gry,M&C,rnd,P srtg,F Si-cem,mchVC,ltl fn;tr glauc & cl
		120-125	5		Ss,Vlt ol gry&gn gry,M&C,rnd,P pyr&lim-cem,ltlVC,mch fn;mch sndy gn gry sh
		125-130	5		Ss,Vlt ol gry&gn gry,M&C,P pyr&lim-cem,ltlVC,mch fn;ltl sndy gn gry sh
		130-145	15	G	Ss,Vlt ol gry&gn gry,M&C,P pyr&lim-cem,trVC,ltl fn&Vfn;tr glauc
		145-150	5	G	Ss,lt ol gry,M&fn,Srnd,P srtg,trVfn,C&VC;tr pyr & glauc
		150-155	5	G	Ss,yl gry,M&C,Srnd,P srtg,tr fn; tr pyr & glauc
		155-165	10	G	Ss,yl gry,M&fn,Srnd,P srtg, ltl Vfn, tr C; tr glauc
		165-170	5		Ss, yl gry, M & fn, Srnd, P srtg, mch Vfn, tr C; tr gry sh
		170-175	5		Ss, lt yl gry, M & fn, Srnd, P srtg, ltl Vfn, tr C; tr gry sh
		175-180	5	G	Ss, lt yl gry, M & C, Srnd, P srtg, VP lim-cem,trVfn&C,ltl fn,tr
		180-185	5	G	Sh,Vlt ol gry,VP Si-cem,ltl Vfn, fn & VC;tr pyr, glauc & sh

Well name
Sample Nos. to

Formation		Depth	Thickness	Lithology	Description
MT SIMON		185-200	15	G	Ss, lt gn gry, M&C, Srnd, P srtg, VP lim-cem, ltlVfn, fn&VC; tr pyr, glauc
		200-210	10	G	Ss, lt gn gry, M&fn, Srnd, P srtg, VP pyr-cem, ltlVfn, fn&VC; tr gn sndy s
		210-215	5	G	Ss, lt gn&ol gry, M&C, Srnd, P srtg, VP pyr-cem; mchVfn, fn&Msnd, trC&VC
		215-225	10	G	Ss, lt ol gry, M&C, Srnd, P srtg, ltl fn&Vfn, trVC; tr pyr, foss&glauc
		225-230	5	G	Ss, lt ol gry, M&fn, Srnd, P srtg, VP lim-cem, ltl Vfn&C; tr glauc&pyr
	242'	230-252	22	G	Ss, lt ol gry, M&fn, Srnd, P srtg, ltl Vfn & C; tr glauc & pyr

END OF WELL

UNIVERSITY OF WISCONSIN GEOLOGICAL & NATURAL HISTORY SURVEY
1815 University Avenue, Madison, Wisconsin 53706

Log No. H63-Tr-68

County: Trempealeau

Well name Blair City Well #5

Owner..... City of Blair
Address... City Hall
Blair, WI 54616
Driller.. Layne-Northwest Co.
Engineer.. Davy Engineering Co.
La Crosse, Wisconsin

Completed... 9/77
Field check.
Altitude.... 845.4'
Use......... Municipal
Static w.l.. 3'3"
Spec. cap... 28 GPM/ft

R. 7 W.
T. 21 N.
Sec. 15

Quad. Blair 15'

Drill Hole						Casing & Liner Pipe or Curbing							
Dia.	from	to	Dia.	from	to	Dia.	Wgt.& Kind	from	to	Dia.	Wgt.& Kind	from	to
72"	0	10'	42"	60'	70'	26"	A53B steel welded	+4'	40'	16"	Stainless steel wire wound screen	45'	70'
66"	10'	23'				16"	A53B steel welded	+6'	45'				
60"	23'	32'											
54"	32'	47'											
48"	47'	60'											

Grout: Kind	from	to
Redi-Mix	0	40'

Samples from 0 to 70' Rec'd: 10/21/77 Studied by: Kathleen Massie Issued: 4/8/82

Formations: Alluvium

Remarks: Well tested for 24 hours at 503 GPM with 17'9" of drawdown.
Well drilled by caisson method.

LOG OF WELL:

	Depths	Graphic Section	Rock Type	Color	Grain Size Mode	Grain Size Range	Miscellaneous Characteristics
ALLUVIUM	0-5		Sand	Yellow	M	Vfn/VC	Trace silt.
	5-10		"	"	"	"	Much gravel(including glauconitic sandstone). Ltl glauconite.
	10-15		"	"	"	"	Little gravel(including glauconitic sandstone). Much glauconite
	15-20		"	"	"	"	Little glauconite. Trace gravel.
	20-25		"	"	Fn&C	"	Same.
	25-30		"	"	M	"	"
	30-35		"	"	"	"	Trace gravel, silt, glauconite.
	35-40		"	"	Fn&C	"	Same.
	40-45		"	Pale yellow	"	"	Trace gravel(including iron oxide cemtd sandstone), st, glauc.
	45-50		"	Yellow	Fn	"	Little glauconite. Trace silt.
	50-55		"	"	M	"	Trace gravel(including sandstone with much mica), glauc., silt.
	55-60		"	"	"	"	Little gravel(including glauconitic ss). Tr silt, glauconite.
	60-65		Gvl & Snd	White	M peb&M	SP/VLP&Vfn/VC	White & Yellow glauconitic sandstone. Tr silt, glauconite.
70'	65-70		Sand	"	M	Vfn/VC	Trace gravel, silt, glauconite.

END OF LOG

UNIVERSITY OF WISCONSIN GEOLOGICAL & NATURAL HISTORY SURVEY
1815 University Avenue, Madison, Wisconsin 53706

Log No H163-Tr-70

Well name Blair City Test Hole #1-76

Owner.... City of Blair
Address.. City Hall
Blair, WI 54616
Driller.. Layne Northwest Co., Inc.
Engineer.

County: Trempealeau

Completed.... 1976
Field check.
Altitude.... 855' ETM
Use......... Test
Static w.l..
Spec. cap...

R. 7 W.
T. 21 N.

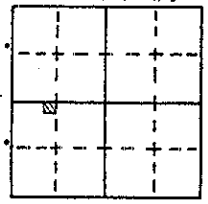

Sec. 15

Quad. Blair 15'

Drill Hole						Casing & Liner Pipe or Curbing							
Dia.	from	to	Dia.	from	to	Dia.	Wgt.& Kind	from	to	Dia.	Wgt.& Kind	from	to

Grout	from	to

Drilling method:
Samples from 0 to 80' Rec'd: 6/16/77

Studied by: Kathleen Massie

Formations: Surface, Alluvium

Remarks:

Issued: 7/16/81

LOG OF WELL:

	Depths	Graphic Section	Rock Type	Color	Grain Size Mode	Grain Size Range	Miscellaneous Characteristics
2' Sfc.	0-2		Soil	V dk bn	—	—	Little organic material.
	2-5		Snd & clay	Strg brown	Fn	Vfn/VC	Much silt. Trace gravel (lim-cem sandstone), caved soil.
A	5-10		Sand	Yl brown	C	"	Mch gvl (lim & hem cem ss). Tr well rnd qtz grans, Fn/M-glauc, st.
L	10-15		"	"	M	"	Trace gravel, well rnd qtz granules, silt, Fn/M glauc.
L	15-20		"	"	Fn/M	"	Same.
U	20-25		"	"	"	"	Same but little gravel.
V	25-30		"	"	"	"	Ltl Fn/M-glauc. Tr gravel (ss), well rounded quartz granules.
I	30-35		"	"	Fn	"	Trace gravel (ss), well rnd quartz granules, Fn/M-glauconite.
U	35-45		"	"	Fn/M	"	Tr well rnd quartz grans, lim-cem ss grans, Fn/M-glauconite.
M	45-50		"	"	M	"	Same.
	50-55		"	"	"	"	Trace gravel (ss), well rnd quartz granules, Fn/M-glauconite.
	55-60		"	Pl yellow	"	"	Same.
	60-65		"	"	"	"	"
	65-70		"	"	"	"	Trace well rnd qtz grans, lim-cem ss grans, Fn/M-glauconite.
	70-75		"	Yl brown	M/C	"	Ltl gvl (lim-cem ss). Tr well rnd qtz grans, Fn/M-glauconite.
78	75-80		"	"	"	"	Same but much gravel.

END OF LOG

WGNHS Well ID 62000207

University of Wisconsin-Extension
Wisconsin Geological & Natural History Survey

DATE 12/9/2015 WELL NAME Hi-Crush Blair PW-B1

LOCATION 725' SW of S. River Rd at Rabbit Run Rd, Preston, WI

COUNTY Trempealeau LOGGED BY PMChase

LATITUDE LONGITUDE

LOCATION METHOD: GPS ☐ AIR PHOTO/TOPO ☐ PLSS ☐ OTHER

ELEVATION 918' ELEVATION METHOD: DEM ☐ TOPO ☐ OTHER

WELL DEPTH 408 CASING DEPTH 260 DEPTH TO WATER 64

CASING STICK UP 1.7 *File Created on:* 2/16/2016 *by:* AMB

Comments: 12" steel casing

LOGS COLLECTED:

Gamma	X	Fluid Conductivity	X
Caliper	X	Flow Meter- HeatPulse	
Single Point Resistivity	X	Flow Meter- Spinner	
Self Potential	X	Optical Borehole Imager	
Normal Resistivity	X	Acoustic Borehole Imager	
Fluid Temperature	X	OTHER:	

Unless Noted:

- *all depths are in feet*
- *well depth, casing depth and depth to water are interpreted from geophysical log*
- *datum is the top of casing*

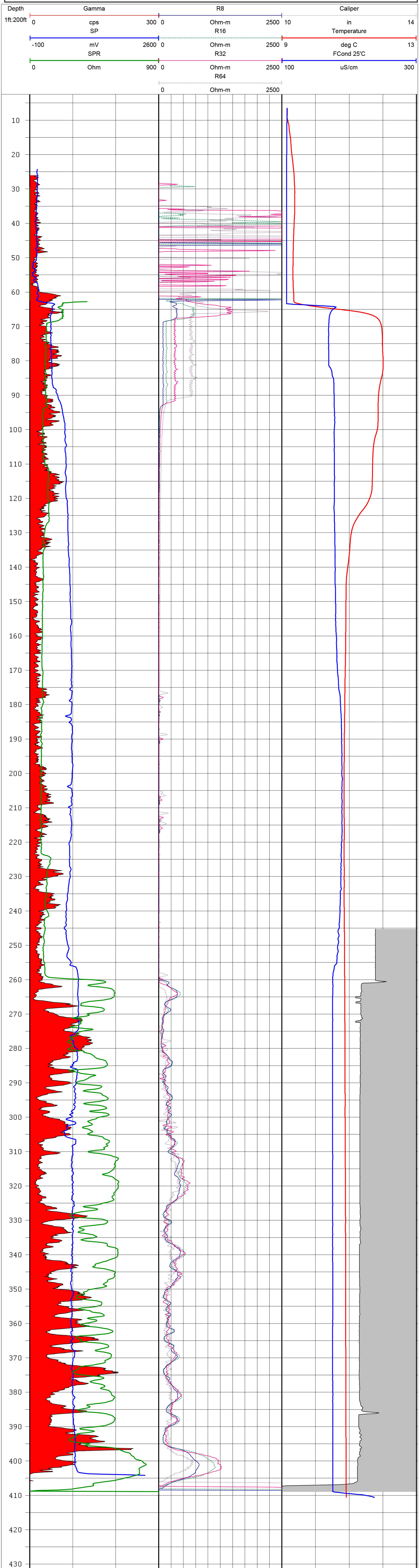

WGNHS Well ID 62000208

UW Extension
University of Wisconsin-Extension
Wisconsin Geological & Natural History Survey

DATE 12/8/2015 WELL NAME Hi-Crush Blair PW-B2
LOCATION 1980' SW of S. River Rd at Rabbit Run Road, Preston, WI
COUNTY Trempealeau LOGGED BY PMChase
LATITUDE LONGITUDE

LOCATION METHOD: GPS ☐ AIR PHOTO/TOPO ☐ PLSS ☐ OTHER
ELEVATION 940' ELEVATION METHOD: DEM ☐ TOPO ☐ OTHER
WELL DEPTH 417 CASING DEPTH 260 DEPTH TO WATER 91
CASING STICK UP 2.2 File Created on: 2/16/2016 by: AMB

Comments: Caliper not calibrated, values are relative.
12" steel casing.

LOGS COLLECTED:

Gamma	X	Fluid Conductivity	X
Caliper	X	Flow Meter- HeatPulse	
Single Point Resistivity	X	Flow Meter- Spinner	
Self Potential	X	Optical Borehole Imager	
Normal Resistivity	X	Acoustic Borehole Imager	
Fluid Temperature	X	OTHER:	

Unless Noted:
- all depths are in feet
- well depth, casing depth and depth to water are interpreted from geophysical log
- datum is the top of casing

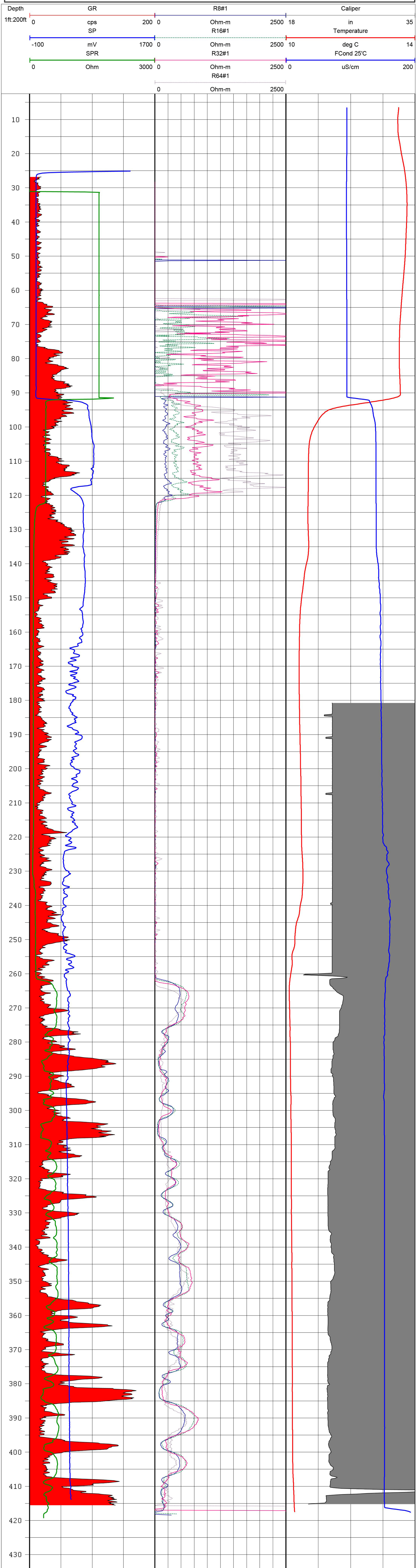

WGNHS Well ID 62000209

UW Extension
University of Wisconsin-Extension
Wisconsin Geological & Natural History Survey

DATE 12/10/2015 WELL NAME PW-B4

LOCATION 1050" SSWof S> River Rd at Rabbit Run Road, Preston, WI

COUNTY Trempealeau LOGGED BY PMChase

LATITUDE LONGITUDE

LOCATION METHOD: GPS ☐ AIR PHOTO/TOPO ☐ PLSS ☐ OTHER

ELEVATION 926' ELEVATION METHOD: DEM ☐ TOPO ☐ OTHER

WELL DEPTH 453 CASING DEPTH 302 DEPTH TO WATER 104

CASING STICK UP 2.0 File Created on: 3/2/2016 by: AMB

Comments: 6" steel casing. Spinner data available.

LOGS COLLECTED:

Gamma	X	Fluid Conductivity	X
Caliper	X	Flow Meter- HeatPulse	
Single Point Resistivity	X	Flow Meter- Spinner	X
Self Potential	X	Optical Borehole Imager	X
Normal Resistivity	X	Acoustic Borehole Imager	
Fluid Temperature	X	OTHER:	

Unless Noted:
- all depths are in feet
- well depth, casing depth and depth to water are interpreted from geophysical log
- datum is the top of casing

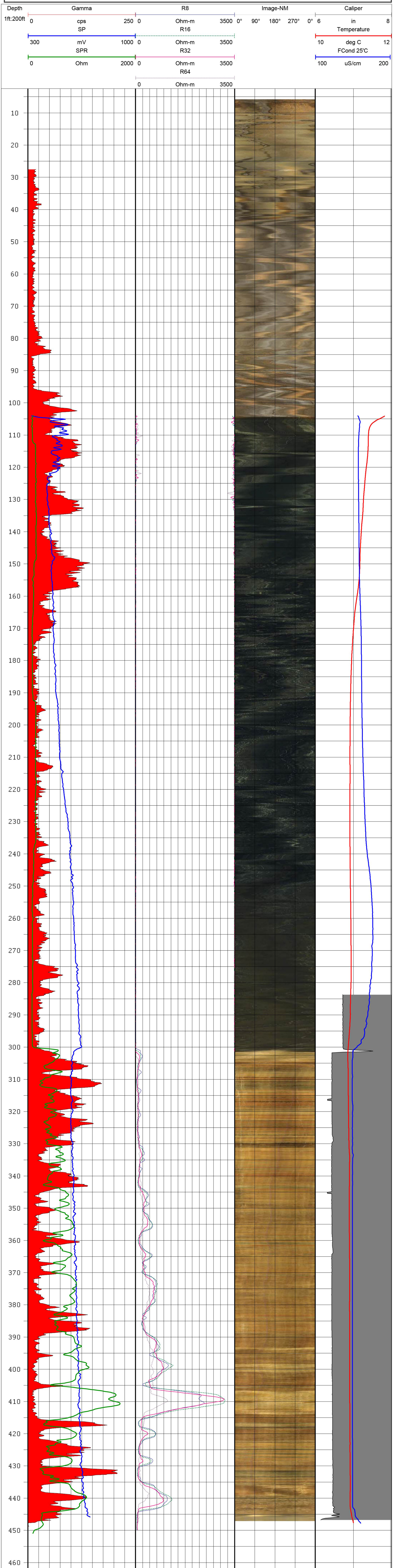

WGNHS Well ID 62000210

UW Extension
University of Wisconsin-Extension
Wisconsin Geological & Natural History Survey

DATE 12/9/2015 WELL NAME Hi-Crush Blair PW-B5
LOCATION 1350' NW of S. River Rd at Rabbit Run Rd, Preston, WI
COUNTY Trempealeau LOGGED BY PMChase
LATITUDE LONGITUDE

LOCATION METHOD: GPS ☐ AIR PHOTO/TOPO ☐ PLSS ☐ OTHER

ELEVATION 871' ELEVATION METHOD: DEM ☐ TOPO ☐ OTHER

WELL DEPTH 332 CASING DEPTH 178 DEPTH TO WATER 46

CASING STICK UP 2.4 File Created on: 2/16/2016 by: AMB

Comments:

LOGS COLLECTED:

Gamma	X	Fluid Conductivity	X
Caliper	X	Flow Meter- HeatPulse	
Single Point Resistivity	X	Flow Meter- Spinner	
Self Potential	X	Optical Borehole Imager	
Normal Resistivity	X	Acoustic Borehole Imager	
Fluid Temperature	X	OTHER:	

Unless Noted:
- all depths are in feet
- well depth, casing depth and depth to water are interpreted from geophysical log
- datum is the top of casing

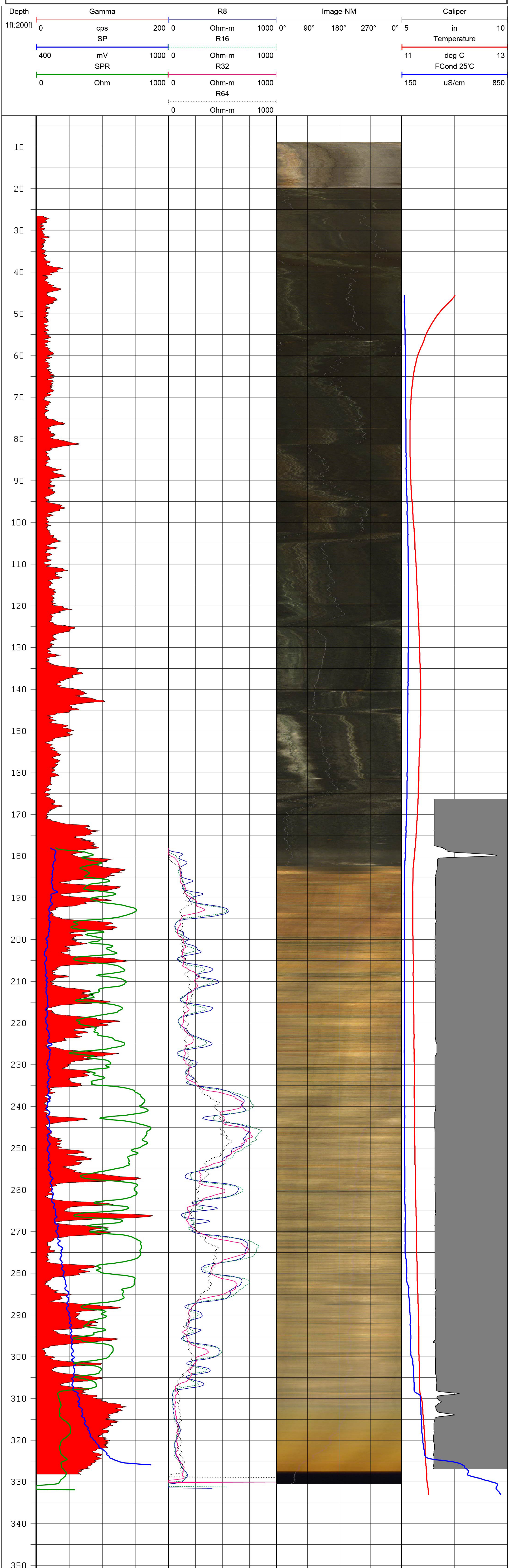

WGNHS Well ID 62000211

UW Extension
University of Wisconsin-Extension
Wisconsin Geological & Natural History Survey

DATE 12/9/2015 WELL NAME Hi-Crush Blair PW-B6
LOCATION 640' WNW of S. River Rd at Rabbit Run Rd, Preston, WI
COUNTY Trempealeau LOGGED BY PMChase
LATITUDE LONGITUDE

LOCATION METHOD: GPS ☐ AIR PHOTO/TOPO ☐ PLSS ☐ OTHER
ELEVATION 892' ELEVATION METHOD: DEM ☐ TOPO ☐ OTHER
WELL DEPTH 352 CASING DEPTH 182 DEPTH TO WATER 40
CASING STICK UP 2.2 File Created on: 2/23/2016 by: AMB

Comments: Well partially obstructed at bottom of casing, OBI and spinner will not pass. Spinner data inconclusive.

LOGS COLLECTED:

Gamma	X	Fluid Conductivity	X
Caliper	X	Flow Meter- HeatPulse	☐
Single Point Resistivity	X	Flow Meter- Spinner	X
Self Potential	X	Optical Borehole Imager	☐
Normal Resistivity	X	Acoustic Borehole Imager	☐
Fluid Temperature	X	OTHER:	☐

Unless Noted:
- all depths are in feet
- well depth, casing depth and depth to water are interpreted from geophysical log
- datum is the top of casing

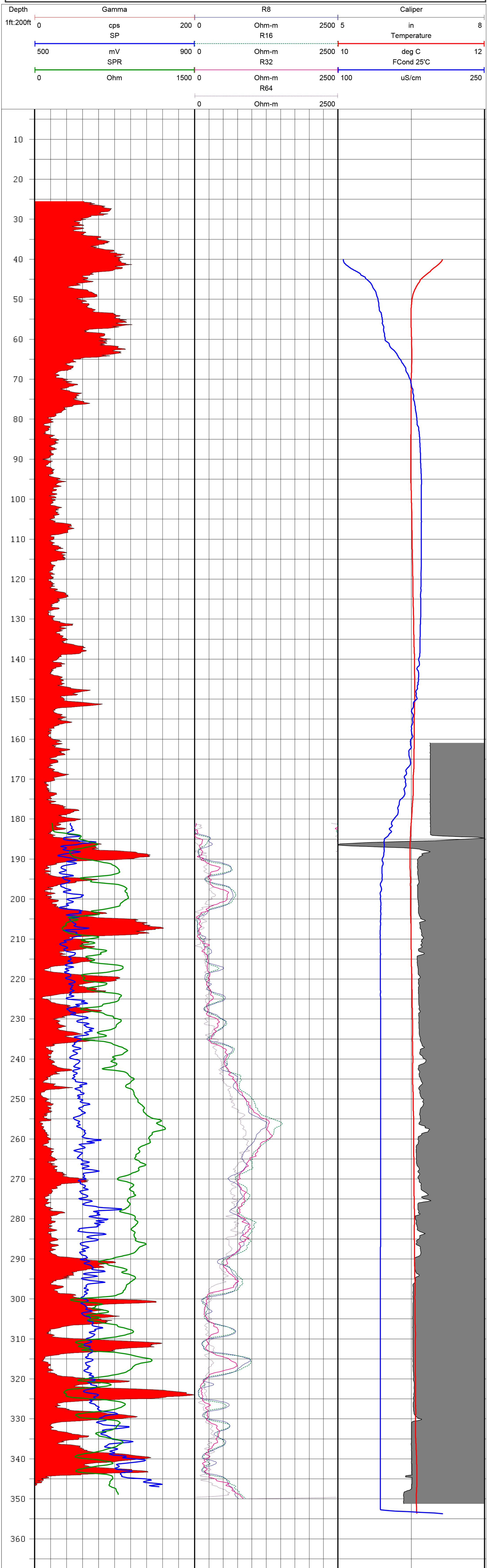

WGNHS Well ID 62000231

University of Wisconsin-Extension
Wisconsin Geological & Natural History Survey

DATE 8/16/2017 WELL NAME WGNHS Kulig
LOCATION 3300 ft W/NW of Witt Hill Rd @ North River Rd., Whitehall
COUNTY Trempealeau LOGGED BY PMChase
LATITUDE 44.37847 LONGITUDE -91.374887
LOCATION METHOD GPS ELEVATION 843' ELEVATION METHOD DEM
WELL DEPTH 212.8 CASING DEPTH 11.5 DEPTH TO WATER 40.2
CASING STICK UP 0.2 WUWN File Created on: 9/19/2017 by: AMB

Comments: *Well Construction field represents the well, casing, and water-level as measured on the day of logging.*

LOGS COLLECTED:

- [X] Gamma
- [ ] Caliper
- [X] Single Point Resistivity
- [X] Self Potential
- [ ] Normal Resistivity
- [ ] Fluid Temperature
- [ ] Fluid Conductivity
- [ ] Flow Meter- HeatPulse
- [ ] Flow Meter- Spinner - flow up is negative, flow down is positive
- [X] Optical Borehole Imager
- [ ] Acoustic Borehole Imager
- [ ] OTHER:

Unless Noted:
- all depths are in feet
- well depth, casing depth and depth to water are interpreted from geophysical log
- datum is the top of casing

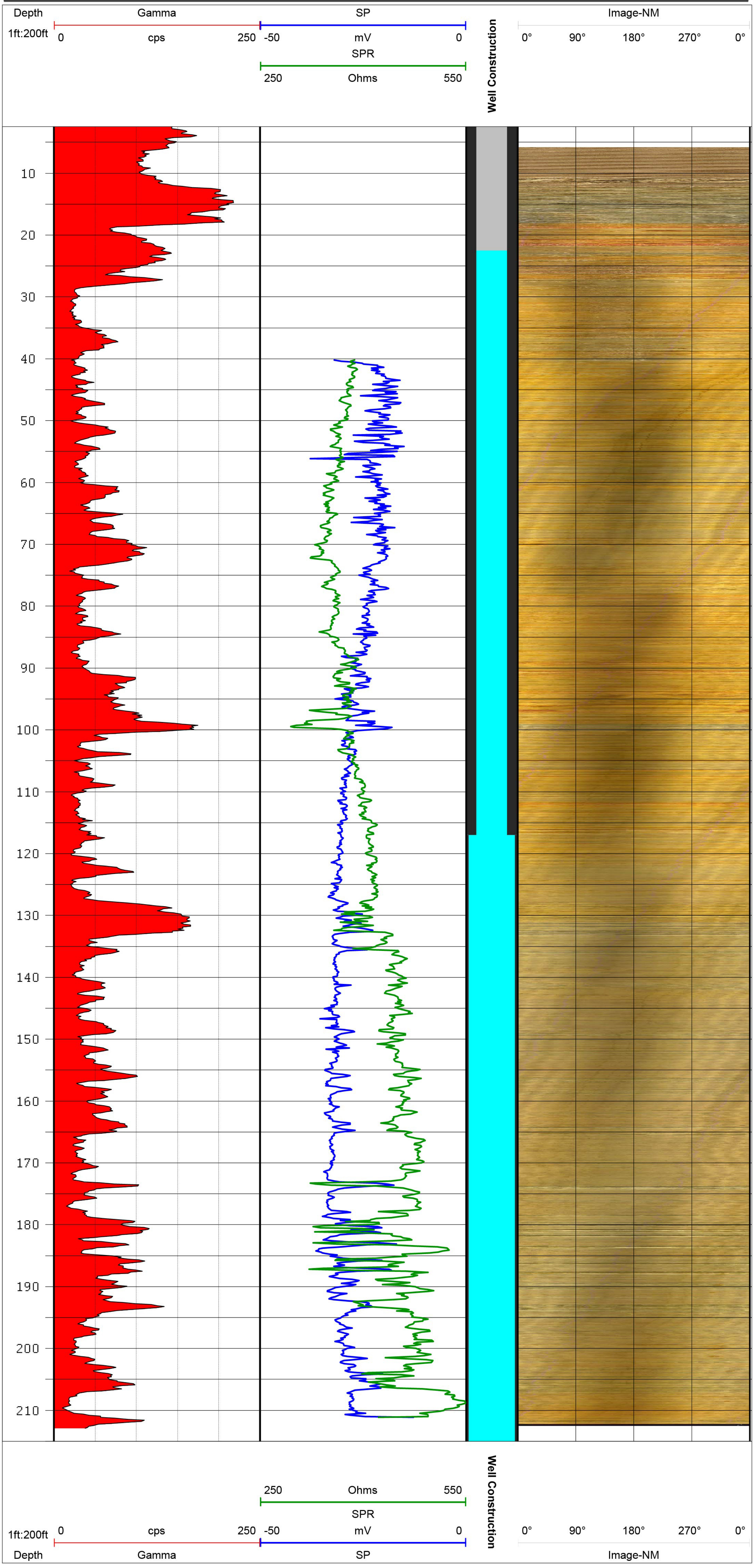

Supplement: Supplementary file 1 [file mmc1.zip › Appendix C_Compilation of Geophysical and Well Construction Information.pdf]
